# Multi‐Stimuli Responsive Viologen‐Crosslinked Eutectogels Derived From Natural Deep Eutectic Solvents for Information Security and Motion Detection

**DOI:** 10.1002/advs.76209

**Published:** 2026-06-19

**Authors:** Zhiang Bai, Wai Lean Koay, Xin Yi Oh, Vinh Xuan Truong

**Affiliations:** ^1^ Institute of Sustainability for Chemicals Energy and Environment (ISCE2) Agency for Science, Technology and Research (A*STAR) Singapore Republic of Singapore; ^2^ Department of Pharmacology Yong Loo Lin School of Medicine National University of Singapore Singapore Republic of Singapore; ^3^ Institute of Materials Research and Engineering (IMRE) Agency for Science, Technology and Research (A*STAR) Singapore Republic of Singapore

**Keywords:** eutectogel, information security, multi‐stimuli responsiveness, natural deep eutectic solvent, photochromism, strain sensing, thermochromism

## Abstract

Stimuli‐responsive soft materials capable of converting environmental and physiological stimuli into optical and electrical signals are highly attractive for information security, wearable sensing, and personalized healthcare management. Herein, we report biocompatible viologen‐crosslinked eutectogels derived from poly(hydroxyethyl acrylate) (poly‐HEA) and natural deep eutectic solvent composed of betaine and lactic acid. The eutectogels exhibit high transparency, excellent elasticity, and thermal stability, as well as robust adhesion on various substrates. The dynamic hydrogen‐bonding network within the network endows the material with outstanding environmental stability and anti‐freezing capability, maintaining stable optical performance under subzero conditions at −20°C. The eutectogels exhibit reversible chromic responses to multiple stimuli including electricity, UV light, and temperature. These optical responses enable reversible photopatterning and multilevel information encryption/decryption. We further demonstrate the functions of the eutectogels as a sensitive and stable strain sensor capable of monitoring diverse human motions, and even subtle physiological heartbeat signals. In addition, the strain‐induced electrical outputs can also be further translated into Morse code for information transmission.

## Graphical abstract

Described herein is the development of an organic eutectic gel based on a deep eutectic mixture of naturally derived betaine and lactic acid. The eutectic gels exhibit reversible color‐switching in response to multiple stimuli including strain, UV light, and temperature, enabling advanced applications in information security, intelligent wearable devices, and advanced healthcare monitoring.

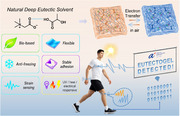

## 1 Introduction

Stimuli‐responsive soft matter materials have seen an enormous resurgence and development in the last 10 years, specifically in the critical areas of personalized healthcare management and information security due to their potential to respond to changes in biometric signals and the environment [1, 2, 3, 4, 5, 6, 7, 8]. These materials are flexible, conductive, often transparent, and are equipped with the capability to detect, memorize, or communicate specific signals in a user‐defined manner. The core mechanism of such advanced functions is the integration of electroactive molecules that change their color upon switching their redox state. Examples of such electrochromic systems include oxides of e.g., tungsten (WO_3_) [9], molybdenum (MoO_3_) [10], titanium (TiO_2_) [11], and bismuth (Bi_2_O_3_) [12], inorganic coordination compounds [13], and organic materials such as phthalocyanines [14], viologens, and conducting polymers [15]. Among those, viologen derivatives have been widely investigated and utilized in flexible electronics due to their distinct color change upon application of a voltage, coupled with excellent reversibility, and stable redox performance. In addition to electrochromism, viologen species also display thermal‐ and photochromism, enabling responsiveness to multiple stimuli generated by the human body or being induced by the surrounding environment e.g., motion, inflammation, or UV light exposure.

Viologen compounds have been incorporated in hydrogel structures for stimuli‐responsive drug release and strain sensing performance [16, 17, 18]. The photo‐switching of the redox states was also utilized for light‐induced actuation of hydroxyethyl acrylate hydrogels, based on the difference in swelling of the hydrogel networks [19]. In addition, viologen‐containing photochromic hydrogels have been used as printing ink on paper materials for anti‐counterfeiting purposes [20]. However, hydrogel materials have low stability in open air due to water evaporation and drying of the materials, leading to the rapid loss of viscoelastic properties and conductivity in the ambient environment. To address this drying issue, viologen‐incorporated ionogels with high ionic conductivity and stable mechanical strength under dry ambient conditions have been developed. Recent examples of such ionogels include poly(ethylene glycol) diacrylate [21], polyvinyl (butyral) [22], poly(acrylamide) [23], or poly(ethyl acrylate) double‐network structures [24] embedded with ionic liquid and viologen species. Viologen‐containing eutectogels using organic solvents such as dimethylformamide [25] or propylene carbonate [21] have also been reported. However, the addition of electrolytes such as 1‐butyl‐3‐methylimidazolium hexafluorophosphate or lithium tetrafluoroborate is necessary for the conductivity and electrochromism function. Despite their promise, these ionogels contain organic solvents and expensive, persistent fluoro‐based ionic liquids that are toxic to humans and harmful to the environment, hampering their potential applications in healthcare management as well as electronic device applications.

Eutectogels are an attractive alternative to ionogels because eutectic solvents can be readily prepared from inexpensive reagents, including hydrogen bond donors (HBD) and hydrogen bond acceptors (HBA) that can also be obtained from natural, renewable sources [26]. The wide selection of HDB and HBA allows for exceptional flexibility in designing eutectogels with tailored physical, chemical, and mechanical properties for specific applications in CO_2_ capture [27, 28], and enhanced drug delivery [29]. While eutectogels have been introduced as strain sensors [30, 31, 32, 33, 34, 35, 36, 37], they have yet to exploit the incorporation of stimuli‐responsive molecules to fully position these materials as an adaptable and multifunctional frontier in advanced sensing, actuation, and therapeutic applications.

**FIGURE 1:**
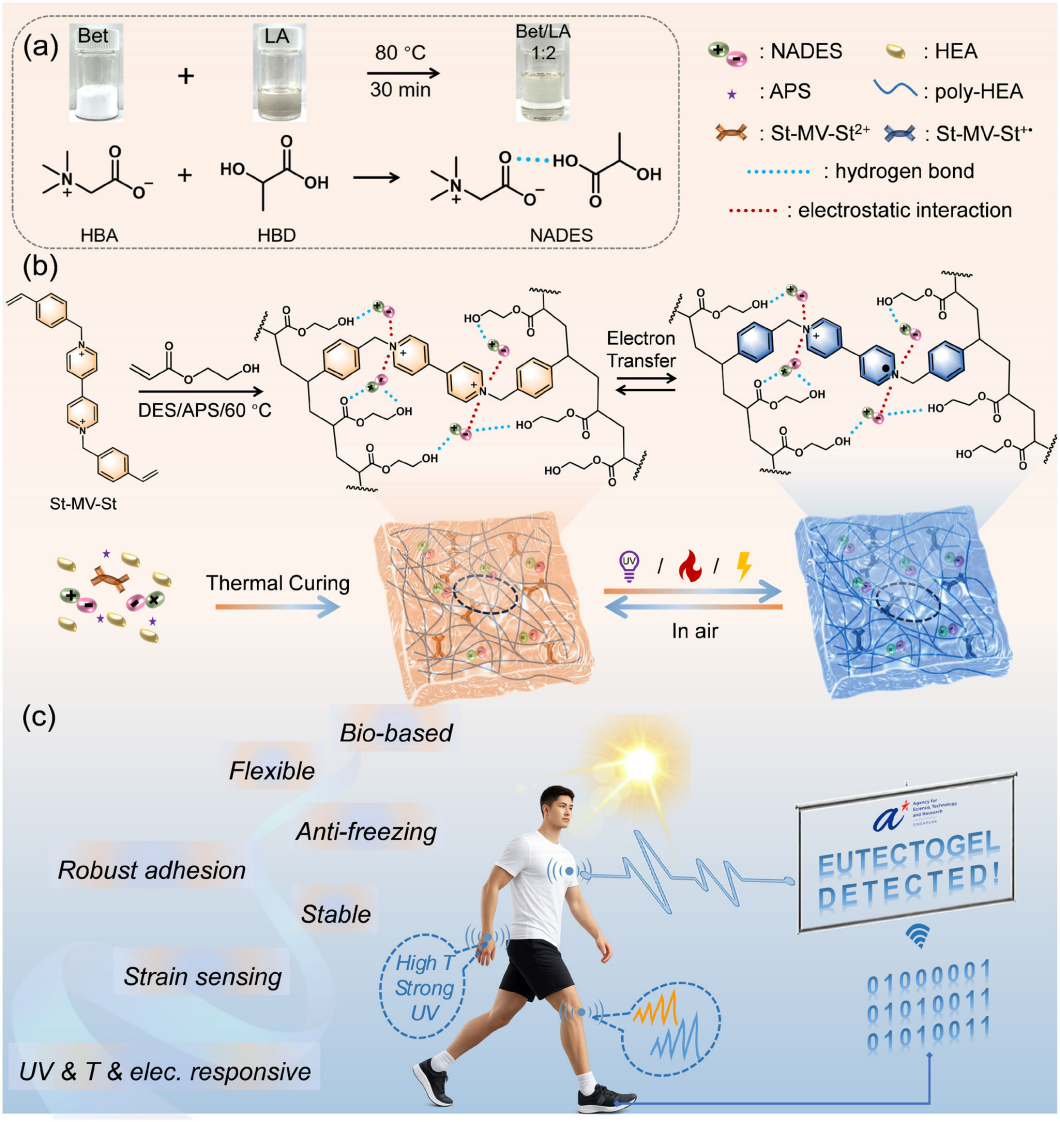
Schematic illustration and application of the eutectogel. (a) Synthetic route of Bet/LA NADES and the images of Bet, LA, and the corresponding NADES at room temperature. (b) Schematic illustration of the synthetic procedure and the network structure of the HSV/DES eutectogels. (c) The performance for HSV/DES eutectogels, and the multi‐stimuli responsive sensing for information security and motion detection.

Our research aims to fill this critical gap by developing a facile method for the fabrication of viologen‐crosslinked polymer structures in a natural deep eutectic solvent (NADES), forming biocompatible eutectogels with multi‐stimuli responsive functions (Figure 1). The low‐cost environmentally friendly NADES is composed of betaine (Bet) and lactic acid (LA)—both have natural origin from living organisms, and the polymer network is synthesized by thermal initiated free‐radical polymerization of hydroxyethyl acrylate (HEA) in the presence of a viologen divinylbenzyl linker (St‐MV‐St) as shown in Figure 1. The resultant eutectogels are stable under ambient conditions and at −20°C, while displaying highly contrast color switching in response to electricity, UV light (λ = 365 nm), and elevated temperature (≥ 100°C), enabling applications in detection of UV light exposure, photo‐patterning, and user‐defined information display. The excellent conductive nature of the eutectogels further allows for highly specific motion‐sensing applications and electrical signaling. This work establishes natural viologen‐crosslinked bio‐based eutectogels as a versatile platform for multifunctional soft devices in information security, motion detection, and wearable healthcare technologies.

## 2 Results and Discussion

### 2.1 Eutectogel Fabrication and Spectroscopic Properties

The bisfunctional styrenic monomer St‐MV‐St was readily synthesized by a one‐step procedure in high yield (Scheme S1) and employed as the crosslinker to prepare eutectogels. The chemical structure of St‐MV‐St was confirmed by NMR spectroscopy (Figure S3). Subsequently, the HSV/DES eutectogels were prepared via thermally initiated free‐radical polymerization by curing a homogeneous mixture of HEA monomer, St‐MV‐St crosslinker, and ammonium persulfate (APS) initiator in Bet/LA NADES at 60°C. This synthesis method overcomes the issues of free‐radical quenching encountered in UV light‐initiation [23]. The resultant samples were henceforth denoted as HSV/DES*_x_* ​, where *x* represents the mass content of NADES. Detailed formulations are summarized in Table 1. To ensure robust gelation and optimal overall performance, the molar ratio of St‐MV‐St relative to HEA was fixed at 0.2 mol% (Table S1 and Figures S4–S6). In addition, eutectogels with various mechanical properties can be obtained by varying the NADES content from 20 to 50 wt.%.

**TABLE 1:** Summary of HSV/DES eutectogels with various ratios of HEA molar content^a^ ^.^

Sample	HSV/DES_20_	HSV/DES_30_	HSV/DES_40_	HSV/DES_50_
NADES (mg)	200	300	400	500
HEA molar content (mmol)	6.81	5.96	5.11	4.25
St‐MV‐St (mmol)	0.0136	0.0119	0.0102	0.0085

^a^ The St‐MV‐St molar content (mol% to HEA) was adjusted at 0.2 mol%, APS initiator was fixed at 0.25 wt.%, and the total mass was maintained at 1000 mg.

As illustrated in Figure 1b, the eutectogel network consists of a covalently crosslinked poly(HEA‐co‐St‐MV‐St) network. During polymerization, hydrophilic HEA units were selected as the monomer because it interacts with the Bet/LA NADES components through hydrogen bond, enabling homogeneous gel formation, efficient NADES retention, flexibility, and uniform dispersion of the St‐MV‐St, which serves as the key crosslinking units for constructing the polymer skeleton. Meanwhile, the NADES synergistically regulates the intermolecular interactions within the eutectogel. The dynamic hydrogen‐bonding network formed between Bet and LA contributes to the flexibility and anti‐freezing capability of the eutectogel, while the NADES also serves as an ion‐conducting medium that facilitates ion migration. In addition, the carbonyl oxygen in the NADES may act as a potential electron donor and participate in the electron transfer (ET) with viologen moieties.

**FIGURE 2:**
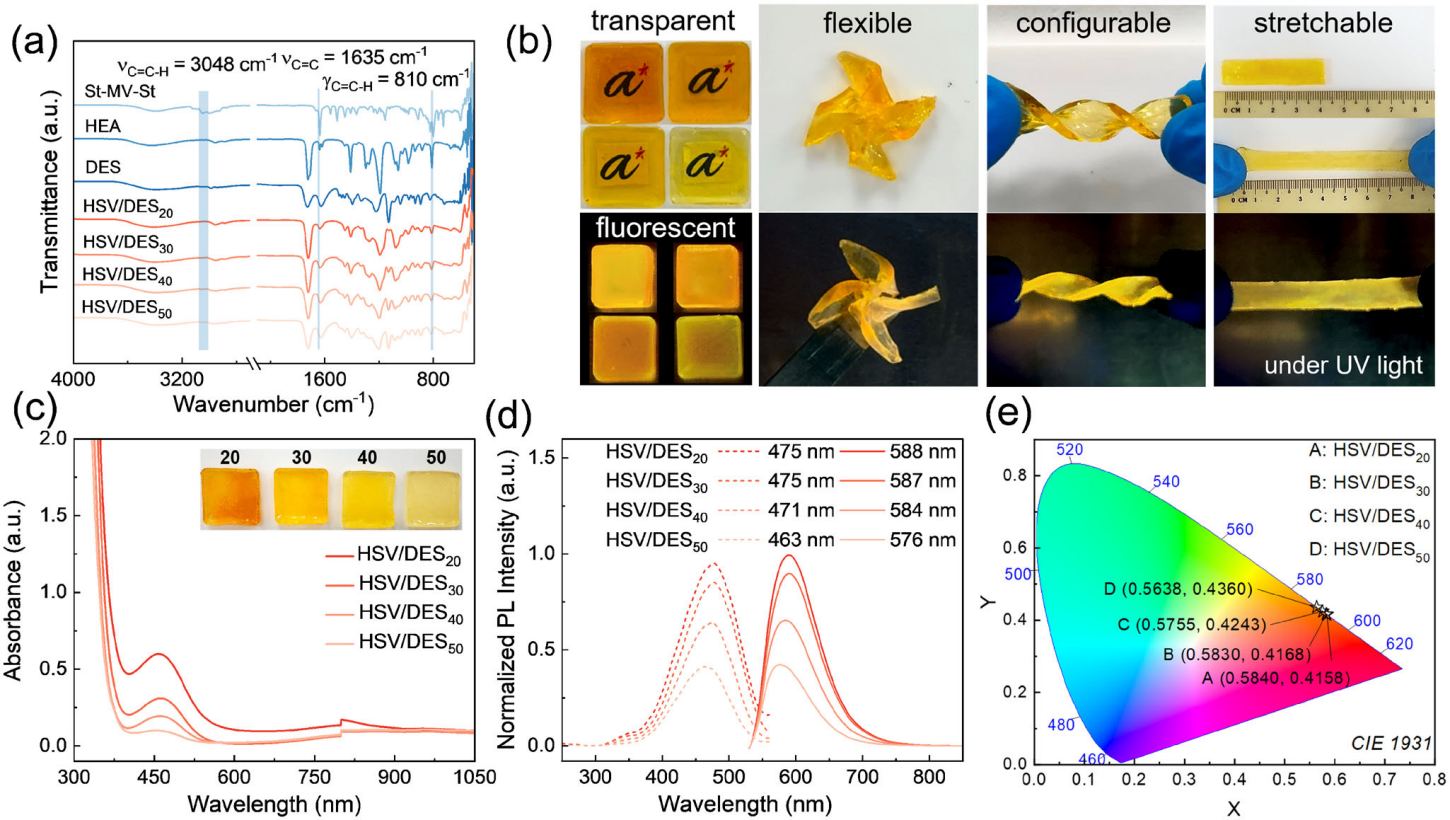
Fabrication and spectroscopic properties of the eutectogel with different NADES mass content. (a) FTIR spectra of St‐MV‐St, HEA, DES, and HSV/DES eutectogels. (b) Photographs illustrating the transparency of HSV/DES eutectogels under ambient light, their fluorescence under UV irradiation, as well as the flexibility, stretchability, and mouldability of the representative HSV/DES_30_ eutectogel. (c) UV–vis spectra of HSV/DES eutectogels, with an inset showing photographs of the corresponding eutectogel. (d) PL spectra of HSV/DES eutectogels. (e) The CIE chromaticity diagrams of PL emission of HSV/DES eutectogels.

The polymerization efficiency of the HEA in HSV/DES eutectogels was first confirmed by FTIR spectroscopy (Figure 2a). Compared with the HEA and St‐MV‐St monomers, the characteristic bands of the carbon‐carbon double bond at 1635, 3084, and 810 cm^−1^ disappear, indicating the completion of polymerization. Moreover, with increasing NADES content, the C═O stretching band gradually shifts from 1725 to 1718 cm^−1^, suggesting strengthened hydrogen‐bonding interactions between the poly(HEA‐co‐St‐MV‐St) network and the NADES components (Figure S7). Consistently, the NMR spectra in Figure S8 reveal that the carboxylic OH signal of LA becomes nearly undetectable due to strong hydrogen bonding between Bet and LA, while the DES‐related resonances progressively shift downfield with increasing NADES content, further supporting the strengthened hydrogen‐bonding interactions [38]. The optical images shown in Figure 2b further confirm the successful fabrication of the eutectogels, which exhibit high transparency and efficient fluorescence. Owing to the dynamic reconfigurability of the hydrogen‐bonding and electrostatic interaction network, the HSV/DES_30_ can be repeatedly reshaped into various temporary shapes, demonstrating excellent flexibility, conformability, and stretchability.

The optical properties of the HSV/DES eutectogels were further investigated by UV–vis and photoluminescence (PL) spectroscopies. As shown in Figure 2c, all samples display a characteristic absorption band at 458 nm, which accounts for their orange color. The absorbance increases with increasing NADES content, consistent with the progressively deepened orange observed in the inset photographs. HSV/DES_30‐50_ reaches high transparency (> 80%) in the visible region, whereas HSV/DES_20_ exhibits relatively lower transmittance as its stronger absorption (Figure S9). Compared to the nearly non‐emissive St‐MV‐St crosslinker, the HSV/DES eutectogels exhibit bright orange fluorescence. The PL intensity of HSV/DES_30_ eutectogel is enhanced by 88.9% relative to that of St‐MV‐St (Figure S10). As shown in Figure 2d, an overall decrease in PL intensity is observed with increasing NADES content, accompanied by slight blue shifts in both the excitation and emission (λ_ex_ from 475 to 463 nm, λ_em_ from 588 to 576 nm), the Commission Internationale de L'Eclairage (CIE) chromaticity coordinates gradually shift from (0.5840, 0.4158) to (0.5638, 0.4360) indicating a slight change in emission color (Figure 2e). The enhanced PL of eutectogels is primarily attributed to the confinement effect imposed by the crosslinked polymer network. St‐MV‐St units are immobilized within the network upon polymerization, which restricts intramolecular rotation and suppresses non‐radiative energy dissipation [39, 40]. Furthermore, incorporation of St‐MV‐St into the polymer also reduces intermolecular aggregation, thereby limiting aggregation‐caused quenching (ACQ) and enhancing PL emission [41]. With increasing NADES content, however, the fluorescence gradually weakens due to the decrease in St‐MV‐St content that lowers the concentration of emissive centers.

### 2.2 Thermal and Mechanical Properties

**FIGURE 3:**
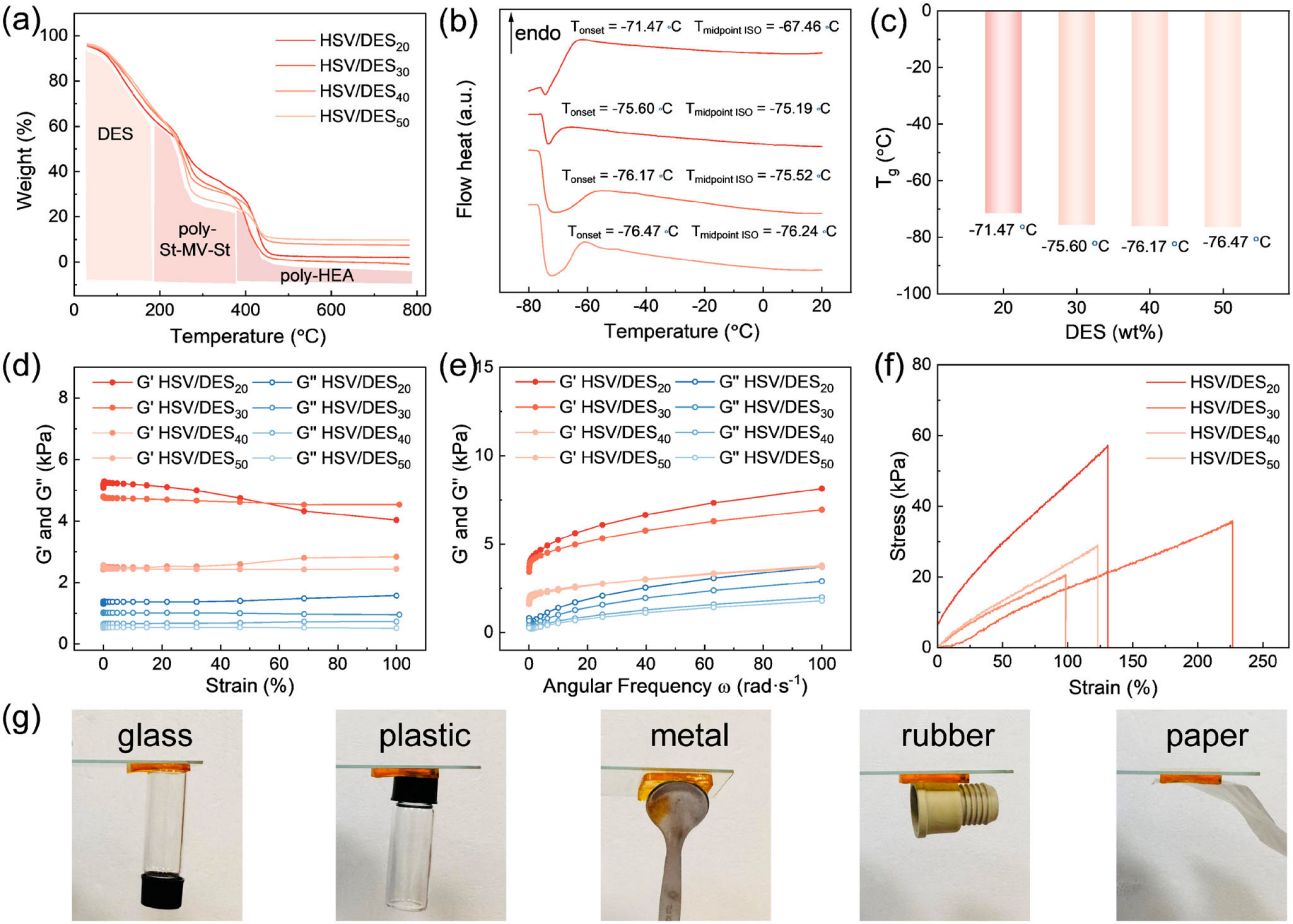
Thermal and mechanical properties of the HSV/DES eutectogels with different NADES mass content. (a) TGA curves of the HSV/DES eutectogels. (b) DSC curves of the HSV/DES eutectogels. (c) T_g_ of the HSV/DES eutectogels. (d) Rheological amplitude sweeps of the HSV/DES eutectogels. (e) Rheological frequency sweep of the HSV/DES eutectogels. (f) Tensile stress–strain curves of the HSV/DES eutectogels. (g) The photos of HSV/DES_30_ eutectogel adhered to various substrates.

The thermal and mechanical properties of the HSV/DES eutectogels were further investigated. The thermal behavior of the eutectogels was characterized by thermogravimetric‐differential thermal analysis (TGA‐DTA) and differential scanning calorimetry (DSC). As shown in Figure 3a, all HSV/DES eutectogels exhibit multistep thermal degradation behavior. The first weight‐loss stage is centred at 110°C for HSV/DES_20_, whereas it shifts to 142 C–158°C for HSV/DES_30–50_, indicating that the DES content significantly affects the initial thermal decomposition behavior of the eutectogels. The subsequent weight‐loss stage at 258 C–262°C is assigned to the decomposition of the St‐MV‐St moieties, which falls within the typical thermal degradation range of viologen species (250 C–400°C) [42]. Further weight loss observed at 399 C–462°C corresponds to the degradation of the poly HEA skeleton of the polymer network (Figures S11–S14). DSC analysis in Figure 3b,c further reveals a gradual decrease in the glass‐transition temperature T_g_ from −71.47 C to −76.47°C with increasing DES content. Notably, HSV/DES_30_ retained its fluorescence after being left in an open‐air environment for 1 month, showing only an 8.9% decrease in PL intensity (Figure S15). Moreover, after storage at −20°C for 1 week, the sample still exhibited excellent flexibility and remained foldable, twistable, squeezable, and stretchable, accompanied by only a 14% loss in PL intensity (Figure S16). These results demonstrate the outstanding environmental stability and excellent anti‐freezing capability of the HSV/DES eutectogels.

The viscoelastic properties of the HSV/DES eutectogels were subsequently investigated by oscillatory rheology. As shown in Figure 3d, the storage modulus (G') remains higher than the loss modulus (G'') over the tested strain range for all samples, confirming the formation of stable gel networks with dominant elastic behavior. Frequency sweeps further show that both G' and G''′ increase slightly with angular frequency, while G' consistently exceeds G'' throughout the measured range (Figure 3e), indicating good structural integrity and viscoelastic stability. Among the samples, HSV/DES_20_ and HSV/DES_30_ exhibit relatively higher moduli, suggesting that moderate NADES incorporation is favorable for maintaining a robust network structure, whereas excessive NADES may dilute the polymer framework and reduce stiffness. The tensile strain is shown in Figure 3f. All samples exhibit good stretchability, while HSV/DES_30_ shows the highest tensile strength of 226.5 kPa. In comparison, HSV/DES_20_, HSV/DES_40_, and HSV/DES_50_ display lower tensile strengths of 130.8, 123.1, and 98.5 kPa, respectively. This composition‐dependent mechanical behavior can be attributed to the balance between the covalently crosslinked polymer skeleton and the dynamic hydrogen‐bonding/electrostatic interactions introduced by the NADES. Considering its balanced optical, thermal, and mechanical performances, HSV/DES_30_ was selected as the representative composition for subsequent investigations. In addition, the eutectogel exhibits good self‐adhesion to various substrates (Figure 3g), coupled with the use of bio‐based materials, offering potential for healthcare applications with direct body contact.

### 2.3 Photochromic Performance

**FIGURE 4:**
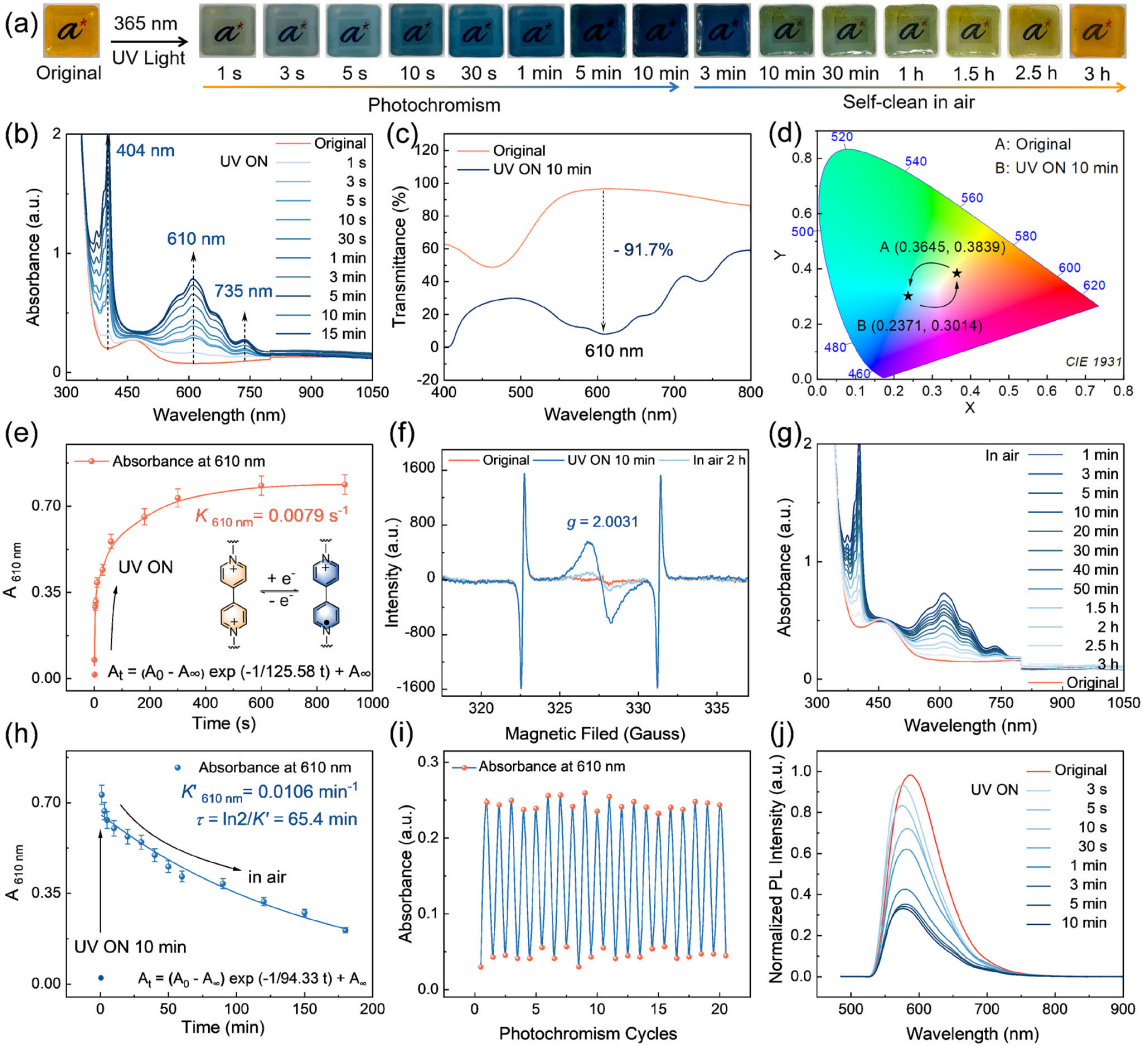
Photochromic performance of HSV/DES_30_ eutectogel. (a) Photographs showing the photochromic process under 365 nm UV irradiation and subsequent self‐cleaning in air at ambient conditions. (Unless otherwise specified, all photochromism experiments were performed under an air atmosphere). (b) UV–vis spectra of HSV/DES_30_ eutectogel recorded at different UV irradiation times. (c) Transmittance spectra of HSV/DES_30_ eutectogel before and after 10 min UV irradiation. (d) The CIE chromaticity diagrams of HSV/DES_30_ eutectogel showing the color change before and after 10 min UV irradiation. (e) The spectral response curve at 610 nm during the photochromic process. (f) ESR spectra of HSV/DES_30_ eutectogel before and after 10 min UV irradiation and in air self‐clean for 2 h. (g) UV‐vis spectra of HSV/DES_30_ eutectogel were recorded during the fading process under ambient conditions for 3 h after 10 min UV irradiation. (h) The spectral decay curve at 610 nm during the fading process. (i) Reversible photochromic cycles of HSV/DES_30_ eutectogel for twenty times. (j) PL spectra of HSV/DES_30_ eutectogel recorded at different UV irradiation times.

The HSV/DES eutectogels exhibit pronounced chromic responses to triple external stimuli including electricity, UV irradiation, and temperature. The electrochromic behavior of St‐MV‐St has already been systematically studied in different polymer systems [43, 44, 45]. As shown in Figure S17, the HSV/DES_30_ eutectogel retained the intrinsic electrochromism of St‐MV‐St, although it's covalently immobilized within the bulk PHEA/NADES network led to a relatively slow electrochromic response [44]. In contrast, photochromism of St‐MV‐St units covalently incorporated into polymer has rarely been reported. Therefore, the photochromic behavior of the HSV/DES_30_ eutectogels was investigated in detail. Upon exposure to 365 nm UV light, the St‐MV‐St crosslinker alone displays only sluggish photochromism (Figure S18). In contrast, the HSV/DES_30_ eutectogel undergoes a rapid and highly contrast color transition from orange to blue within only 1 s, reaching a saturated state after 10 min of UV irradiation (Figure 4a). The UV–vis spectra were recorded to monitor the photochromic process of HSV/DES_30_ under different UV irradiation times. As shown in Figure 4b, a sharp absorption peak appears at 404 nm together with a broad absorption band spanning 450–800 nm, which can be attributed to the formation of viologen radicals under UV irradiation [46]. After 10 min of irradiation when the color reaches saturation, the transparency of HSV/DES_30_ decreases by 91.7% relative to the original state (Figure 4c). Correspondingly, the CIE chromaticity coordinates shift from (0.3645, 0.3839) to (0.2371, 0.3014), consistent with the color change observed (Figure 4d). To further quantify the rapid photochromic process, the apparent rate constant (*K*) was determined to be 0.0079 s^−1^ by monitoring the absorbance change at 610 nm according to the kinetic model (Figure 4e and Figure S19). Electron spin resonance (ESR) measurements further confirmed the generation of radicals during the photochromic process (Figure 4f). No ESR signal was detected before UV exposure, whereas a characteristic nitrogen‐free radical signal with a *g* value of 2.0031 appeared after UV irradiation. IR spectra verify that the gel structure remains intact after UV irradiation (Figure S20), further suggesting that the chromic behavior originates from photoinduced radical formation rather than isomerization or photocatalysis.

The photochromic HSV/DES_30_ eutectogel gradually recovers to its initial state in ambient atmosphere, requiring approximately 3 h for complete decoloration (Figure 4a,g). The radical lifetime was further evaluated from the decay curve of the absorbance at 610 nm (Figure 4h). Based on the kinetic model (Figure S21), the decay rate constant (*K*’) is calculated to be 0.0106 min^−1^, corresponding to a half‐life (*τ*) of 65.4 min. Consistent with the fading behavior, the ESR spectra in Figure 4f show that the radical signal almost disappears after being in air for 2 h. The photochromic cyclic test of HSV/DES_30_ demonstrates excellent reversibility and cycling stability (Figure 4i and Figure S22). Moreover, the HSV/DES_30_ eutectogel exhibits a dynamic decrease in PL intensity during the photochromic process. At the saturated colored state, the PL intensity is quenched by 66.3%. Upon fading, the PL signal gradually recovers showing a corresponding upturn in emission intensity (Figure S23).

The photochromic mechanism can be rationalized based on the donor‐acceptor (D‐A) architecture constructed within the gel network. Viologen species are well‐known electron acceptors, whereas carbonyl groups are relatively electron‐rich and can serve as electron donors. Previous studies have demonstrated that the carbonyl‐containing part can undergo photoinduced intermolecular ET with pyridine nitrogen under UV irradiation [47, 48]. In the HSV/DES eutectogel system, the introduction of Bet/LA NADES components together with the HEA monomer enriches the polymer network with abundant C═O groups that act as electron donors and form an efficient D‐A system with St‐MV‐St [41]. The crosslinked polymer network also brings donor and acceptor units into close spatial proximity, facilitating efficient ET. Upon UV irradiation, rapid intermolecular ET occurs, generating radical cations that are responsible for the blue coloration. The reversed oxidation process can be induced by atmospheric oxygen. Indeed, the color fading in a nitrogen atmosphere is significantly slower than that in open air (Figure S24), indicating that oxygen efficiently quenches the viologen radicals [49]. Therefore, the presence of oxygen accelerates radical oxidation and facilitates the recovery of the original state, enabling a “self‐clean” process under ambient conditions.

### 2.4 Photopatterning and Information Encryption/Decryption

**FIGURE 5:**
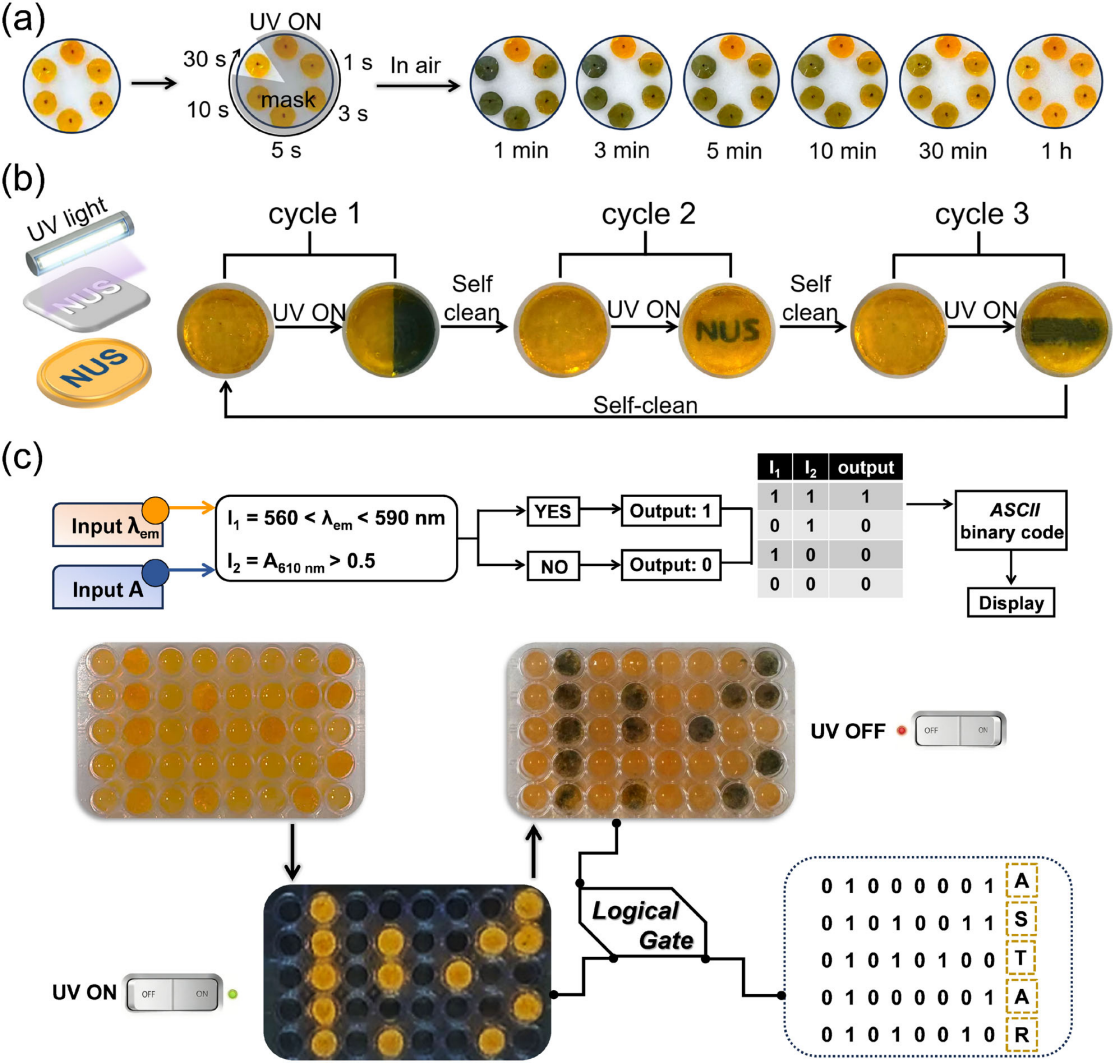
Reversible photopatterning and multilevel information security platform using photo‐responsive HSV/DES_30_ eutectogel. (a) Photographs showing the dynamic color change under different UV irradiation times and self‐cleans in air at ambient conditions. (b) Schematic representation of photopatterning and continuous reversible photopatterning cycles. (c) Optical logic gate for multilevel information encryption and decryption based on absorbance and PL wavelength signals.

Direct photopatterning is an emerging alternative patterning strategy that utilizes photosensitive materials and shows great potential for applications in information transmission and security [50, 51]. The excellent photo‐responsive behavior of our eutectogels holds significant promise for complex data encryption. As a proof‐of‐concept demonstration in Figure 5a, a circular dot pattern was fabricated on HSV/DES_30_ eutectogel. Upon UV irradiation for different times, the pattern developed distinct shades of blue corresponding to its photochromic behavior, showing a user‐defined temporal control of color switching. By using a mask to control the irradiation time, the color depth of the pattern can be precisely tuned, enabling dynamic pattern generation. Notably, the pattern gradually fades and completely self‐cleans within 1 h under ambient conditions. To demonstrate dynamic photopatterning, the HSV/DES_30_ was exposed to UV light via patterned masks (Figure 5b). In cycle 1, partial irradiation induced a rapid color change from orange to blue in the exposed regions, while the masked areas remained unchanged. After removing the UV light, the blue area gradually self‐cleans in the air due to oxidation of the viologen radicals. The same gel was then re‐patterned using different masks. In cycle 2, the letters “NUS” appeared upon UV irradiation and disappeared through the same self‐cleaning process. In cycle 3, a horizontal stripe pattern was generated and subsequently faded after removing the light stimulus. These results demonstrate that HSV/DES_30_ enables reversible and repeatable photopatterning without additional chemical treatment. The pattern can be repeatedly written‐erased through simple UV irradiation and air exposure, highlighting potential for dynamic information encryption.

Conventional optical encryption typically relies on a single optical signal [52], however, HSV/DES_30_ integrates dual optical inputs to construct a logic gate for information encoding. As illustrated in Figure 5c, input I_1_ is defined as the emission signal within the wavelength range of 560–590 nm, while input I_2_ corresponds to the absorbance at 610 nm generated from the photochromic viologen radicals. When both conditions are satisfied (I_1_ = 1 AND I_2_ = 1), the logical output is defined as “1” otherwise is “0”. HSV/DES_30_ eutectogel array initially appears uniform under UV OFF conditions, giving no readable information. Upon UV ON, both PL and photochromic responses generate spatially resolved optical signals that can be converted into binary sequences through the logical gate operation. According to the ASCII (American Standard Code for Information Interchange) scheme, the resulting binary codes can be decoded into characters to display the encrypted message “ASTAR”.

### 2.5 Thermochromic Performance

**FIGURE 6:**
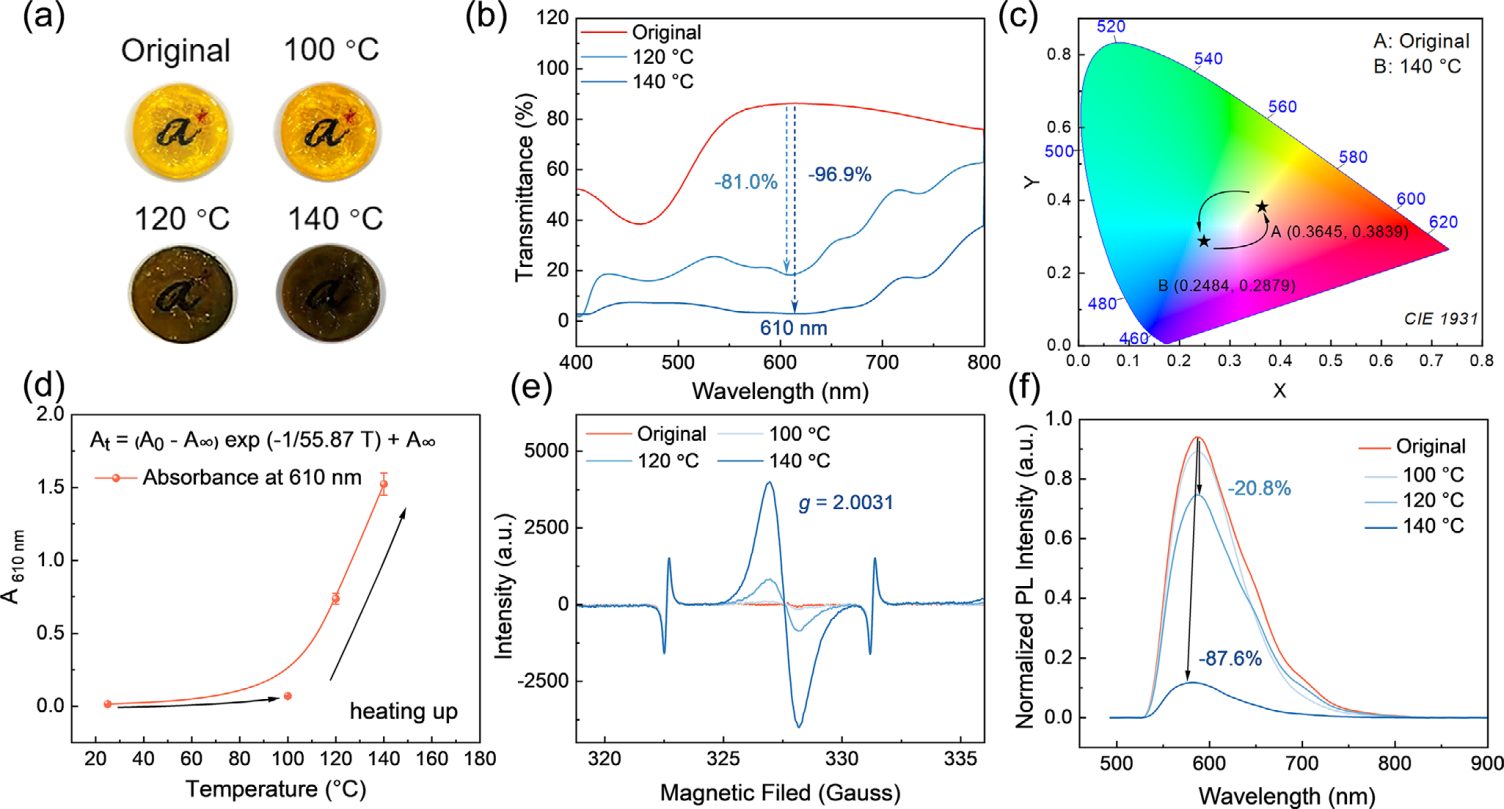
Thermochromic performance of HSV/DES_30_ eutectogel. (a) Photographs showing the thermochromic process under different temperatures. (b) Transmittance spectra of HSV/DES_30_ eutectogel recorded at before and after heating to 120°C and 140°C. (c) The spectral response curve at 610 nm during the thermochromic process. (d) The CIE chromaticity diagrams of HSV/DES_30_ eutectogel showing the color change before and after heating to 140°C. (e) ESR spectra of HSV/DES_30_ eutectogel before and after heating to different temperatures and in air self‐cleans for 2 h. (f) PL spectra of HSV/DES_30_ eutectogel recorded before and after heating to different temperatures.

HSV/DES_30_ eutectogel also exhibits sensitive and reversible thermochromic behavior while St‐MV‐St itself shows no response to heat, indicating the thermochromism does not originate from the viologen unit alone. Before gelation, St‐MV‐St freely dispersed in the NADES medium and can interact efficiently with the electron‐efficient NADES; therefore, thermally induced ET occurs with an obvious color change observed at 60°C (Figure S25). After gelation, St‐MV‐St is covalently incorporated into the PHEA/NADES network and becomes immobilized within the polymer matrix. The restricted molecular mobility and surrounding PHEA network reduce the contact and ET efficiency between St‐MV‐St and NADES components [44], thus requiring higher thermal energy to generate sufficient viologen radicals. Therefore, HSV/DES_30_ eutectogel exhibits thermochromism at elevated temperatures above 100°C. As discussed previously, the HSV/DES_30_ gel will lose NADES when the temperature exceeds approximately 142°C. Therefore, the thermochromic behavior was investigated within a temperature range up to 140°C. As shown in Figure 6a, the HSV/DES_30_ eutectogel displays a brownish color when heated to 120°C, which further deepens at 140°C. Similar to the photochromic response, the thermal‐induced color change is reversible and can be quenched by oxygen, recovering to its original state upon exposure to ambient air. Consistent with these visual observations, UV–vis spectroscopy reveals a pronounced decrease in optical transparency, with reductions of 81% at 120°C and 96.9% at 140°C (Figure 6b). The UV–vis spectra in Figure S26 also show the emergence of broad absorption bands spanning 300–800 nm, which can be attributed to the formation of free radicals primarily generated through intermolecular electron transfer between the electron‐donating NADES components and the electron‐deficient viologen units [53]. The CIE chromaticity diagram reveals color shift from (0.3645, 0.3839) to (0.2484, 0.2879) after heating to 140°C. Although the CIE coordinates shift toward the blue region, the samples appear brown to the naked eye. This discrepancy arises from the substantial decrease in overall transparency and enhanced broadband absorption, which reduce the transmitted light intensity and alter the perceived color. The apparent rate constant (*K*) of the thermochromism was determined to be 0.0179°C^−1^ by monitoring the absorbance at 610 nm according to the kinetic model (Figure 6d and Figure S27). The generation of free radicals was further confirmed by ESR test, showing a signal at *g* = 2.0031 at 120°C. The ESR signal intensity increases markedly at 140°C, indicating a rapid increase in radical concentration with increasing temperature (Figure 6e). This can be attributed to the abundant carbonyl groups in the Bet/LA NADES, which can serve as electron‐donating sites and interact with the electron‐deficient St‐MV‐St under thermal stimulation [41]. To further verify the essential role of the Bet/LA NADES in the ET process, control experiments were performed by replacing NADES with DMSO/water while keeping rest unchanged. The DMSO‐ and water‐based gels exhibited no obvious thermal color change (Figure S28), indicating that thermochromism does not simply originate from thermal treatment alone. Instead, the specific Bet/LA NADES is crucial for promoting thermally induced ET and stabilizing the generated radicals. Notably, the radical formed at 120°C gradually fades and returns to its original state within approximately 3 h, demonstrating the reversibility of the thermochromic process (Figure S29). PL spectra show a trend consistent with the thermochromic behavior (Figure 6f), as temperature increases, the gel color becomes darker while the PL intensity decreases. At 120°C, the PL intensity decreases by 20.8%. Upon further heating to 140°C, the gel becomes significantly darker and the PL intensity decreases by 87.6% relative to its initial value. These properties position HSV/DES_30_ eutectogel as a promising candidate for practical applications in stimuli‐responsive materials.

### 2.6 Strain Sensing Performance

**FIGURE 7:**
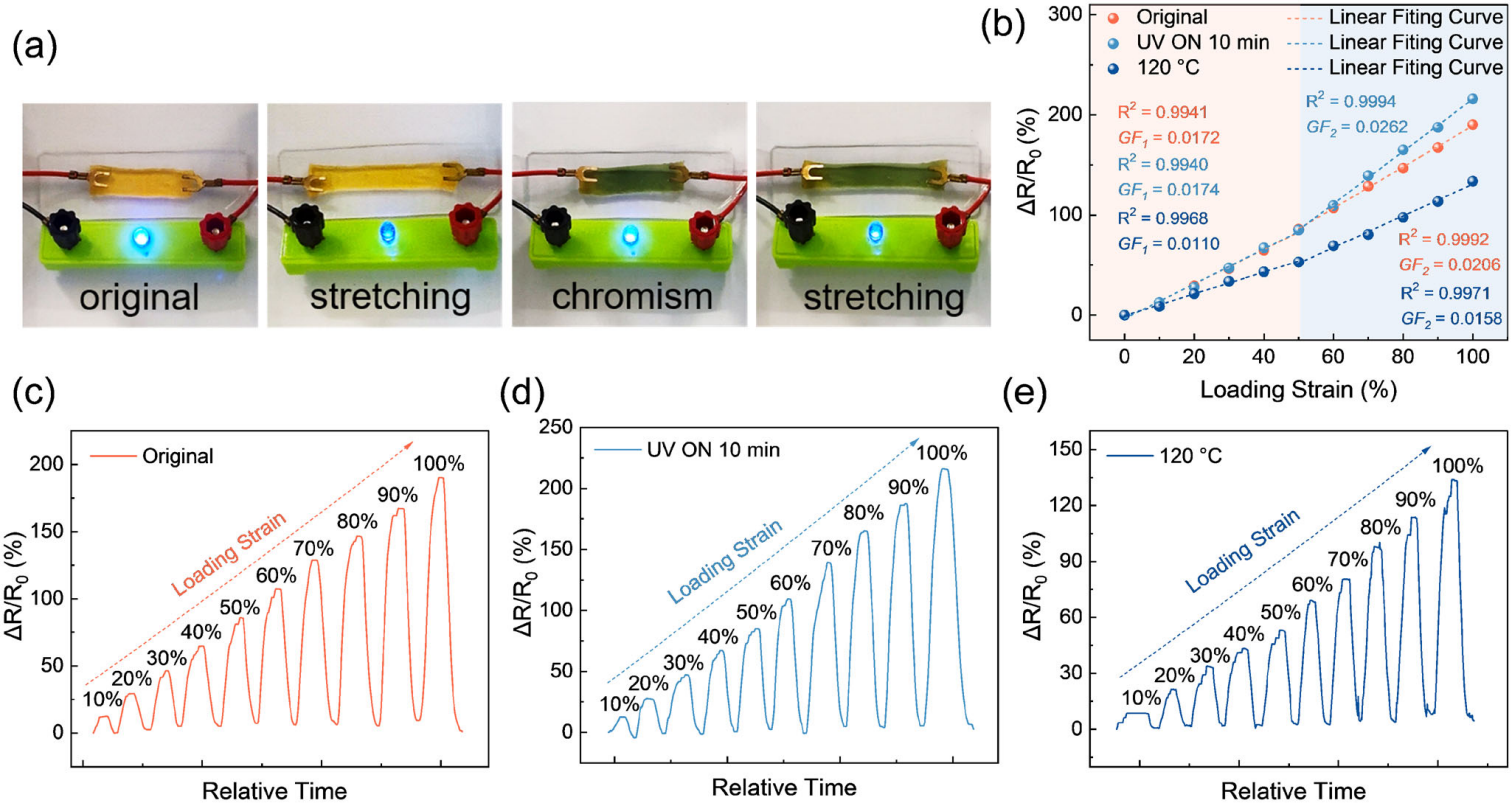
The strain‐sensing performance of HSV/DES_30_ eutectogel. (a) Photographs showing the change in light intensity of the LED bulb during the stretching and chromism process. (b) Variation of relative resistance of HSV/DES_30_ eutectogel with different loading strain before and after 10 min UV irradiation and heating to 120°C. (c) Relative resistance variation of HSV/DES_30_ eutectogel with different loading strains from 10%–100%. (d) Relative resistance variation of HSV/DES_30_ eutectogel after 10 min UV irradiation with different loading strain from 10%–100%. (e) Relative resistance variation of HSV/DES_30_ eutectogel after heating to 120°C with different loading strains from 10%–100%.

The intrinsic conductivity of the HSV/DES_30_ eutectogel enables its application in flexible sensing devices. As shown in Figure 7a, a blue LED bulb in an open circuit can be illuminated when connected to the HSV/DES_30_ eutectogel. The brightness of the bulb varies upon stretching, indicating strain‐dependent electrical conductivity of the material. Notably, a change in brightness can also be observed before and after the chromic process, demonstrating that the HSV/DES_30_ eutectogel can function as a multi‐stimuli‐responsive chromic strain sensor.

To further evaluate its strain‐sensing performance, a resistive strain sensor was fabricated using the HSV/DES_30_ eutectogel. The sensitivity of the sensor was quantified by the gauge factor (GF), defined as the relative resistance change (ΔR/R_0_) with respect to the applied strain. As shown in Figure 7b, the HSV/DES_30_ eutectogel exhibits a two‐stage linear resistance‐strain relationship with high correlation coefficients. In the low‐strain region (0%–50%), the GF values of the original, photochromic (UV ON for 10 min), and thermochromic (120°C) states are 0.0172, 0.0174, and 0.0110, respectively. The comparable GF values between the original and photochromic states with a slightly lower value for the thermochromic state, indicate that chromic transitions have minimal influence on strain sensing under small deformation. In contrast, at higher strain levels (50%–100%), the photochromic state exhibits the highest GF value of 0.0262, compared with 0.0206 and 0.0158 for the original and thermochromic states, respectively. This result suggests that photochromic activation enhances strain sensitivity under larger tensile deformation.

Fast response and recovery behaviors are also observed during the loading‐unloading processes. The response times of the original, photochromic, and thermochromic states are 0.41, 0.35, and 0.43 s, respectively, while the corresponding recovery times are 0.47, 0.31, and 0.35 s (Figures S31–S33). To further assess sensing reliability, the ΔR/R_0_ was monitored under various strains ranging from 10% to 100%. As shown in Figure 7c–e and Figures S34–S36, the eutectogel exhibits stable and repeatable resistance responses during cyclic stretching, demonstrating excellent electromechanical stability. Additionally, the eutectogel demonstrated rapid and consistent electrical response across various applied stretching strain. There is no obvious signal fluctuation of ΔR/R_0_ at different strain, which indicates the signal stability (Figures S37–S38). These findings highlight the ability of the eutectogel sensor to effectively monitor different strains and generate real‐time targeted feedback electrical signals.

### 2.7 Multi‐Stimuli Sensing for Motion Detection and Information Transmission

**FIGURE 8:**
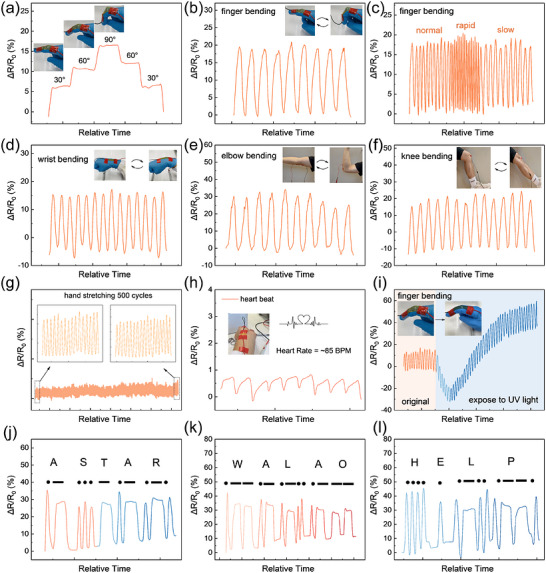
The multi‐stimuli sensing performance of HSV/DES_30_ eutectogel for motion detection and information transmission. (a) Relative resistance changes during consecutive finger bending with different angles. (b) Relative resistance changes during consecutive finger bending. (c) Relative resistance changes during consecutive finger bending with different bending speeds. (d) Relative resistance changes during consecutive wrist bending. (e) Relative resistance changes during consecutive elbow bending. (f) Relative resistance changes during consecutive knee bending. (g) Relative resistance changes during consecutive hand stretching for 500 cycles. (h) Relative resistance changes when monitoring the heartbeat. (i) Relative resistance changes during consecutive finger bending before and after exposure to UV light. (j) Strain sensing for information transmission by Morse code and output signals as “ASTAR”, (k) “WALAO”, and (l) “HELP”.

Stimuli‐responsive strain sensors based on the HSV/DES_30_ eutectogel were fabricated to monitor various human motions in real time. As illustrated in Figure 8a, the eutectogel strip was attached to the finger joint to detect finger bending. The sensor exhibited clear and stepwise increases in relative resistance with increasing bending angles, accompanied by stable and repeatable electrical signals of ΔR/R_0_ around 5%, 10%, and 15% for sequential bending angles of 30°, 60°, and 90° (Figure 8a,b). When the finger returned to its initial position, the resistance recovered to its original level, demonstrating excellent sensing reversibility. The high sensitivity of the eutectogel sensor also enabled the detection of different bending speeds. As shown in Figure 8c, normal, rapid, and slow bending motions produced electrical signals with different frequencies while maintaining consistent amplitudes, allowing real‐time monitoring of motion dynamics. The sensor was further applied to monitor other human motions. When attached to the wrist or elbow, the device effectively detected bending motions and produced distinct resistance responses corresponding to the deformation of the joints (Figure 8d,e and Movies S1 and S2). Similarly, the sensor can track knee movements during walking or leg lifting, generating stable electrical signals for both slow and rapid motions (Figure 8f). In addition to joint bending, the sensor also enables continuous monitoring of hand stretching. As shown in 8 g and Figures S39–S40, repetitive hand stretching induces highly reproducible and stable resistance variations over than 500 times cycles even exposed in open air after 3 months, the eutectogel still maintained its integrity and stretchability, confirming its robustness and stability under cyclic tensile deformation. Furthermore, as shown in Figure 8h, the gel sensor can monitor human heartbeat signals, producing a detectable relative resistance change of approximately 1.5% (Movie S3). This result demonstrates the high sensitivity of the sensor and highlights its potential for applications in personalized healthcare monitoring.

Moreover, the HSV/DES_30_ eutectogel enables coupling between chromic response and strain sensing. Figure 8i shows the photochromism as an illustration, the resistance signal during finger bending exhibits a distinct response after UV irradiation due to the photochromic activation. The resistance signal initially decreases and shows a short buffering stage, followed by a rapid increase during bending deformation. This unique signal evolution can be attributed to the formation of photoinduced radicals, which modulate the local charge distribution around St‐MV‐St sites and induce short‐term ion redistribution. This process may temporarily buffer the resistance change and cause the initial resistance decrease. The buffer and recovery stage lasts for approximately 30 s, consistent with the rapid radical generation during photochromism. As bending proceeds, strain‐induced deformation of ion‐transport pathways becomes dominant, leading to the subsequent rapid resistance increase. Such chromic‐strain sensing coupling provides an additional signal modulation mechanism for the sensor, which is more appliable in practical life. Furthermore, the electrical signals generated by repeated bending motions can be translated into Morse code, enabling simple information encoding through mechanical stimuli. The strain sensor was utilized to output English words, including “ASTAR”, “WALAO”, and “HELP” as shown in Figure 8j–l, demonstrating that the output signals exhibit high readability, supporting the potential application of the HSV/ DES_30_ eutectogel in motion detection, information transmission, and human–machine interaction. Compared with other stimuli‐sensing materials that use strain sensors with visible readouts (Table S2), the present eutectogel moderate sensing sensitivity and stability, while simultaneously enabling rapid stimuli responses, reversible information encryption, and strain‐sensing capability within one NADES‐based soft platform, demonstrating its potential as a multifunctional smart responsive material.

## 3 Conclusion

In conclusion, we present a biobased viologen‐crosslinked eutectogel platform derived from a natural deep eutectic solvent that integrates optical/thermal responsiveness and electromechanical sensing within a single soft material system. The eutectogel combines high transparency, flexibility, and robust thermal and mechanical stability with excellent environmental tolerance, maintaining stable optical performance during long‐term storage and retaining flexibility even at −20°C. Critically, the donor‐acceptor eutectic solvent system within the polymer network enables efficient stimulus‐triggered electron transfer, enabling reversible photochromic and thermochromic responses. These optical behaviors support dynamic photopatterning and multilevel information encryption. Furthermore, the conductive eutectogel network provides reliable and sensitive strain sensing, allowing monitor of diverse human motions and physiological signals. Importantly, the strain‐induced electrical signals can be encoded into Morse code, enabling mechanical‐signal‐based information transmission. We submit that our natural eutectogels hold significant potential as a multifunctional soft platform for information security, intelligent wearable devices and advanced healthcare monitoring.

## Funding

R25J1IR019 and IAF‐PP: H23J2a0130 from Agency for Science, Technology and Research (A*STAR) Singapore.

## Conflicts of Interest

The authors declare no conflicts of interest.

## Supporting information




**Supporting File 1**: advs76209‐sup‐0001‐SuppMat.docx.

**Supporting File 2**: advs76209‐sup‐0002‐VideoS1.mp4.

**Supporting File 3**: advs76209‐sup‐0003‐VideoS2.mp4.

**Supporting File 4**: advs76209‐sup‐0004‐VideoS3.mp4.

## Data Availability

The data that support the findings of this study are available from the corresponding author upon reasonable request.
