# Supplementary material for: Multi‐Stimuli Responsive Viologen‐Crosslinked Eutectogels Derived From Natural Deep Eutectic Solvents for Information Security and Motion Detection
Source: Adv Sci (Weinh). 2026 Jun 19:e76209. Online ahead of print. doi: 10.1002/advs.76209 (PMC13336943; doi:10.1002/advs.76209)
Supplement: Supplementary file 1 — Supporting File 1: advs76209‐sup‐0001‐SuppMat.docx. [file ADVS-9999-e76209-s002.docx]

Supporting Information

Multi-Stimuli Responsive Viologen-Crosslinked Eutectogels Derived from Natural Deep Eutectic Solvents for Information Security and Motion Detection

Zhiang Bai^1,2^, Wai Lean Koay^1^, Xin Yi Oh^1^, Vinh Xuan Truong^1,2,3^*

*Corresponding author: Vinh Xuan Truong

*^1^Institute of Sustainability for Chemicals, Energy and Environment (ISCE2), Agency for Science, Technology and Research (A*STAR), 1 Pesek Road, Jurong Island, Singapore 627833, Republic of Singapore

^2^Department of Pharmacology, Yong Loo Lin School of Medicine, National University of Singapore, Singapore 117600, Republic of Singapore

^3^ Institute of Materials Research and Engineering (IMRE), Agency for Science, Technology and Research (A*STAR), 2 Fusionopolis Wy, Singapore 138634, Republic of Singapore*

*E-mail:* [*Vinh_Truong@a-star.edu.sg*](mailto:Vinh_Truong@a-star.edu.sg)

**Table of Content**

[**1. Materials Synthesis 3**](#_Toc224051739)

[**2. Characterization Methods 5**](#_Toc224051740)

[**3. Supplementary Figures 8**](#_Toc224051741)

[**4. Supplementary Videos 25**](#_Toc224051742)

[**5. Reference 25**](#_Toc224051743)

# 1. Materials Synthesis

All the chemicals and solvents (AR-grade reagents) were obtained from commercial sources and are used as received without any further purification. Specifically, 4,4'-bipyridine (98% purity) was obtained from BLD Pharmatech; 4-vinylbenzyl chloride (purity > 99%) was supplied by Fluka; anhydrous N, N-dimethylformamide (99.8% purity), ammonium persulfate (98% purity), betaine (purity ≥ 98%), DL-lactic acid (purity > 85%), and acetone (ACS reagent, purity ≥ 99.5%) were purchased from Sigma Aldrich; 2-hydroxyethyl acrylate (HEA), stabilized with MEHQ (purity > 95%), was obtained from Tokyo Chemical Industry (TCI). The betaine-based NADES (Bet/LA) and crosslinker monomer 1,1-bis-((4-vinyl)benzyl)-4,4'-bipyridine dichloride salts (St-MV-St) were synthesized according to the reported literature procedures.^[1, 2]^

**Synthesis of Bet/LA NADES.** The mixtures were prepared by mixing betaine with lactic acid at molar ratios of 1:2, mixture was placed in a tightly sealed glass bottle, stirred continuously using a magnetic stirrer and heated at 80 ℃ in an oil bath for 30 min until a clear, homogenous liquid was formed.

**Synthesis of St-MV-St Crosslinker.** Under 90% nitrogen atmosphere, 4-chloromethyl styrene (4 eq.) and 4,4’-bipyridine (1 eq.) were dissolved in anhydrous dimethylformamide (DMF) in a round-bottom flask. After heating at 60 ℃ for 24 h, the reaction mixture was filtered out. The filtrate was washed with anhydrous acetone, yielding a yellowish solid powder after drying under vacuum at R.T. (4.2 g, yield: 82%). ^1^H NMR (400 MHz, D_2_O) δ (ppm) 9.16 (d, J = 7.0 Hz, 4H), 8.53 (d, J = 7.0 Hz, 4H), 7.61 (d, J = 8.4 Hz, 4H), 7.51 (d, J = 8.3 Hz, 4H), 6.81 (m, 2H), 5.92 (m, 4H), 5.89 (s, 2H), 5.38 (d, J = 10.9 Hz, 2H).


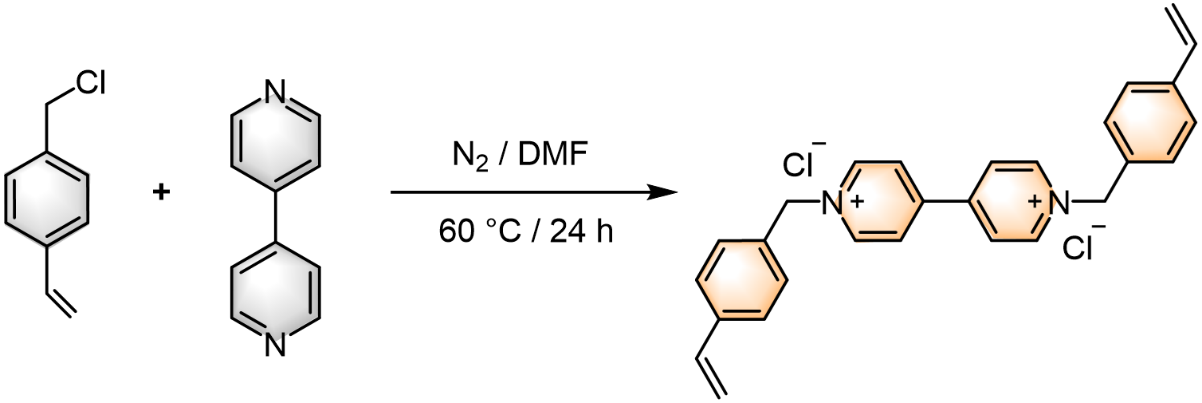


**Scheme S1.** The synthetic route of St-MV-St molecule.

**Eutectogel fabrication.** St-MV-St (0.2 mol% relative to HEA monomer) was dissolved in HEA monomer, followed by vortexing for 30 s to ensure homogeneous mixing and the formation of a clear solution. Different weight percentage Bet/LA NADES (20, 30, 40, and 50 wt%) was then added to the HEA and St-MV-St solution, then ammonium persulfate (APS, 0.25 wt%) was subsequently introduced as the initiator. The mixture was vortexed for another 30 s to achieve uniform dispersion. The resulting precursor solution was then transferred to a square Teflon mold and thermally cured in an oven at 60 °C for 20 min to obtain the orange-colored eutectogel sample. The cured eutectogel samples were then carefully removed from the mold using a spatula. All eutectogel samples subjected to spectral measurements were prepared as square specimens with dimensions of 2 × 2 × 0.2 cm, as illustrated in the Figure S2. Larger eutectogel samples for testing and photopatterning contained 30 wt% NADES fabricated using 10 times the amount of material listed in Table S1.


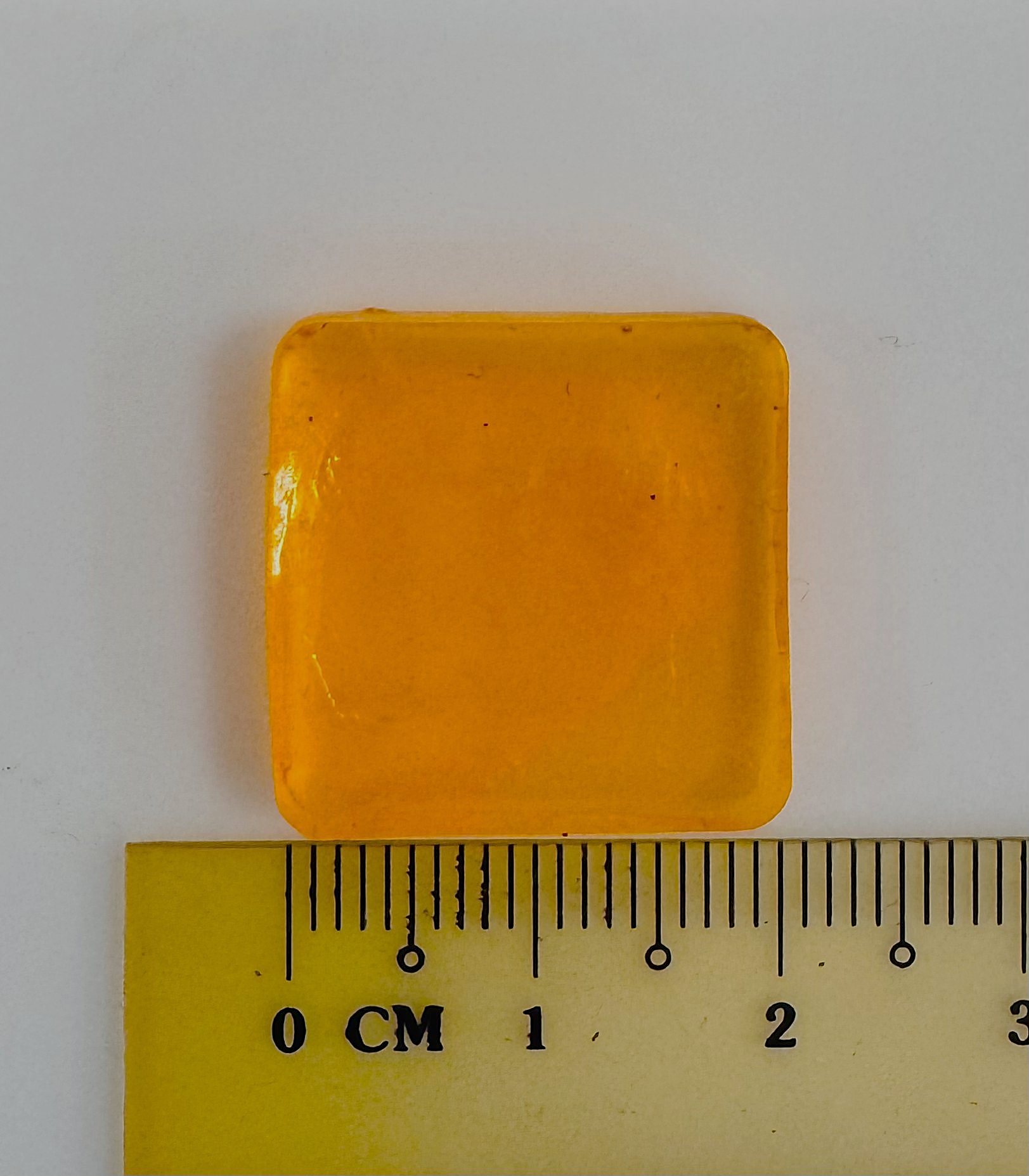


**Figure S1.** Representative photo of an eutectogel sample.

| **Table S1.** General protocol for eutectogel preparation with different crosslinker content^b^ | | | | |
| --- | --- | --- | --- | --- |
| Sample | HSV_0.1_/DES_30_ | HSV_0.2_/DES_30_ | HSV_0.3_/DES_30_ | HSV_0.4_/DES_30_ |
| NADES (wt%) | 30 | | | |
| HEA (mg) | 694.7 | 692.0 | 689.3 | 686.5 |
| HEA molar content (mmol) | 5.98 | 5.96 | 5.94 | 5.91 |
| St-MV-St (mg) | 2.8 | 5.5 | 8.2 | 10.9 |
| St-MV-St relative to HEA (mol%) | 0.1 | 0.2 | 0.3 | 0.4 |

^b^ The NADES mass content was fixed at 30 wt%, the APS initiator was fixed at 0.25 wt%, and the total mass kept at 1000 mg.

# 2. Characterization Methods

**Nuclear Magnetic Resonance (NMR)** spectra were recorded on a Bruker spectrometer (400 MHz) at ambient temperature. D_2_O (Cambridge Isotope Laboratories, USA) were used as the solvents for the NMR analysis, and the chemical shift was calibrated using residual undertreated solvents or tetramethyl silane (TMS) as the internal standard.

**Fourier transform infrared (FTIR)** spectra were recorded on a Bruker α II Platinum ATR-FTIR spectrometer coupled with a HYPERION 3000 accessory, with 32 scans accumulated at a spectral resolution of 4 cm⁻¹.

**UV-Vis** **Spectra** were recorded on Agilent Cary 5000 UV-Vis-NIR Spectrophotometer equipped with Tungsten halogen visible and deuterium arc UV. Calculation details of responsivity and decay rate. The calculated responsivity rate is according to the reaction kinetics equation:

ln[(A_0_−A_∞_)/(A_t_−A_∞_)] = *K*·t

where A_0_ and A_∞_ are the initial and saturated absorbance respectively, *K* is the responsivity rate constant, and t and A_t_ are the irradiation time and the corresponding absorbance. This formula can evolve into:

A_t_ = (A_0_−A_∞_) × e^(-^*^K^* ^t)^ + A_∞_

The calculated decay rate and half-life period are according to the reaction kinetics equation:

ln[(A_0’_−A_∞’_)/(A_t’_−A_∞’_)] = *K*’·t’

τ = ln2/*K*’

where A_0’_ and A_∞’_ are the saturated and completely quenched absorbance respectively, *K*’ is the decay rate constant, t’ and A_t’_ are the quenching time and the corresponding absorbance, τ is the half-life period. This formula can evolve into:

A_t’_ = (A_0’_−A_∞’_) × e^(-k’t’)^ + A_∞’_

**Photoluminescence (PL) Spectra** were recorded on Agilent Cary Eclipse fluorescence spectrometer with Xenon flash lamp (80 Hz). Samples were measured in quartz high precision slides at ambient temperature.

**Electron Spin Resonance (ESR)** measurements were performed on a JEOL JES-X3 ESR spectrometer. The magnetic field was calibrated using the characteristic hyperfine splitting signals of a manganese (II) oxide standard marker. The prominent peaks labeled m1 (322.633 mT, g=2.03390) and m2 (331.310 mT, g=1.98063) in the spectra correspond to the Mn²⁺ reference signals, which were utilized for the precise determination of the g-factors of the sample signals. These Mn²⁺ calibration peaks were not associated with the sample signal.

**Figure S2.** Blank scanning with two Mn markers.

**Thermogravimetric Analysis and Differential Thermal Analysis (TGA-DTA)** measurement were recorded on TA Instruments TGA Q500 under a nitrogen atmosphere. Samples (~5-6 mg) were heated from 30 to 800 °C at a rate of 10 °C min^-1^ to evaluate the thermal stability and decomposition behavior of the eutectogel.

**Differential Scanning Calorimetry (DSC)** were recorded on SDT 650-1 DSC/DTA thermal analyzer under a nitrogen atmosphere. Samples (≈ 3-6 mg) were sealed in indium pans and subjected to a heating-cooling cycle from 20 °C to −80 °C at a rate of 10 °C min^-1^.

**Rheological experiments** were conducted using an Anton Paar Physical rheometer with a plate-plate configuration. The lower plate is made of quartz and the upper plate is made of stainless steel with a diameter of 25 mm.

**Tensile tests** were recorded on a universal testing machine (Instron model 5569), equipped with a 1 kN load cell at a speed of 50 mm/min, using pneumatic side action tensile grips. Tensile stress and strain of the specimens were recorded by the Bluehill 3 software.

**Strain sensing** tests were conducted using a Keithley DMM6500 6½-digit bench/system digital multimeter (Tektronix, USA), which features a high-precision 2-wire resistance measurement mode with a broad measurable range of 1 mΩ to 100 MΩ and is integrated with a high-resolution real-time clock (15 ns timestamp resolution) for accurate time-series data acquisition.

Light Source of the UV light irradiation on the eutectogel samples was performed using commercial light-emitting diode (LED) lamps (15 W) with irradiance centered at 365 nm, and the intensity was calibrated at I = 20 mW·cm^−2^. The International Commission on Illumination (abbreviated CIE for Commission internationale de l'éclairage) is used CIE 1931 for graphs. All the photo and images were captured using the camera of iPhone 15 Pro Plus.

# 3. Supplementary Figures


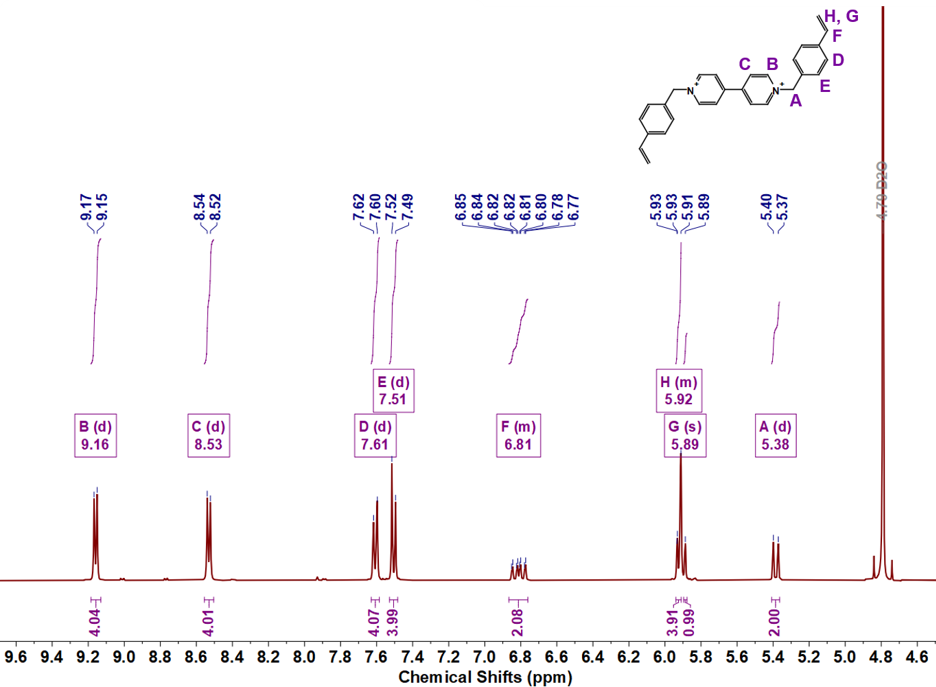


**Figure S3.** NMR spectra of St-MV-St (400 MHz, D_2_O).

**Figure S4.** FTIR spectra of St-MV-St, NADES and HSV/DES eutectogel with different crosslinker content

**Figure S5.** UV-Vis spectra of HSV/DES_30_ eutectogel with different crosslinker content.

**Figure S6.** PL spectra of HSV/DES_30_ eutectogel with different crosslinker content. (λ_ex_ = 475 nm, B.W. = 2.5 nm)

**Figure S7.** FTIR spectra of DES and HSV/DES eutectogel.


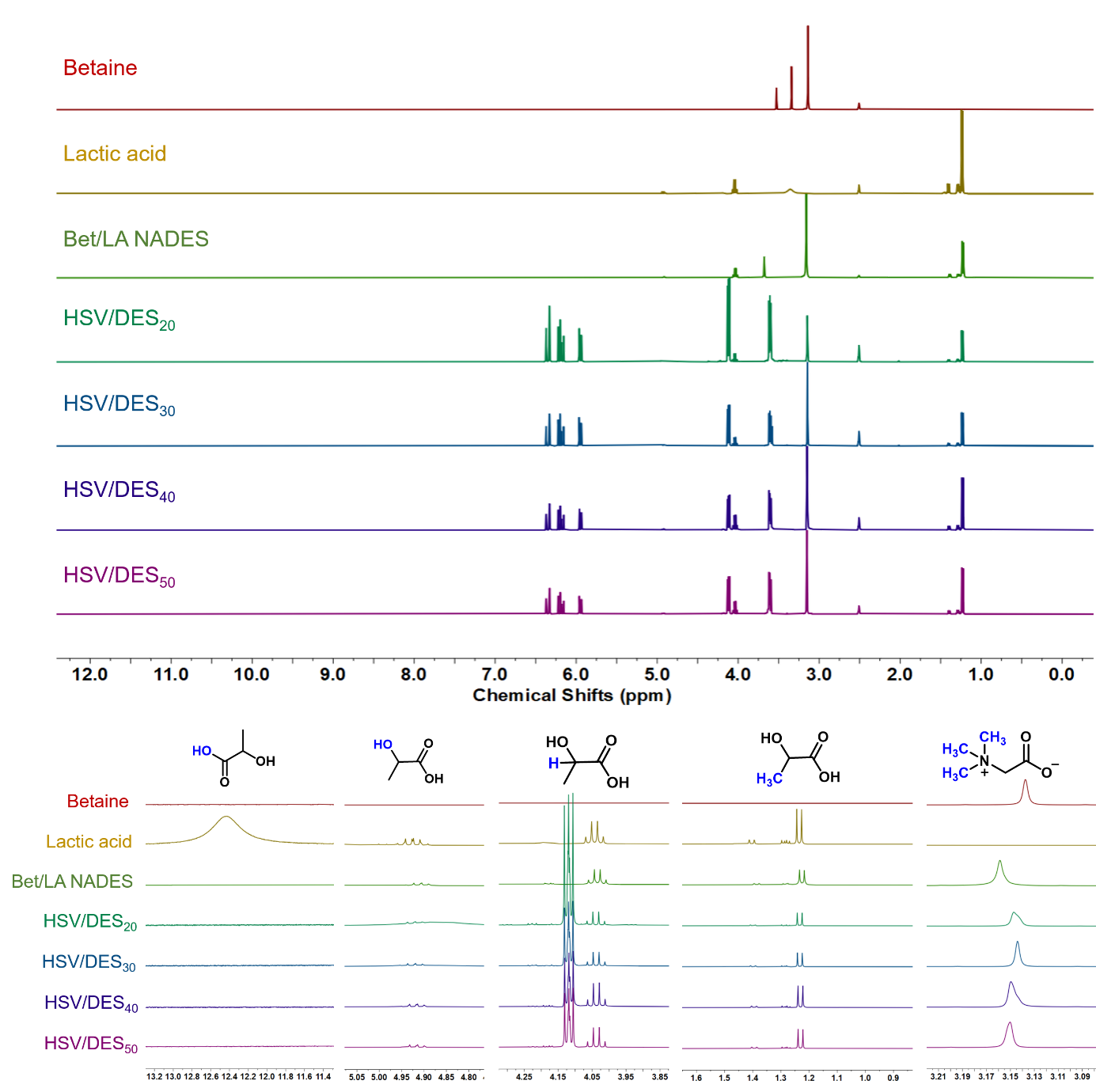


**Figure S8.** NMR spectra of betaine, lactic acid, Bet/LA NADES, and HSV/DES eutectogels with different NADES mass contents before polymerization. The lower panels show the enlarged regions corresponding to the characteristic proton signals. (400 MHz, d6-DMSO).

**Figure S9.** Transmittance spectra of eutectogel with NADES of 20, 30, 40 and 50 wt%.

**Figure S10.** PL spectra of St-MV-St solid (λ_ex_ = 401 nm, λ_em_ = 548 nm, B.W. = 2.5 nm) and HSV/DES30 eutectogel (λ_ex_ = 475 nm, λ_em_ = 587 nm, B.W. = 2.5 nm).

**Figure S11.** TGA-DTA spectra of HSV/DES_20_ eutectogel.

**Figure S12.** TGA-DTA spectra of HSV/DES_30_ eutectogel.

**Figure S13.** TGA-DTA spectra of HSV/DES_40_ eutectogel.

**Figure S14.** TGA-DTA spectra of HSV/DES_50_ eutectogel.

**Figure S15.** PL spectra of HSV/DES_30_ eutectogel after 1 month (λ_ex_ = 475 nm, B.W. = 2.5 nm).


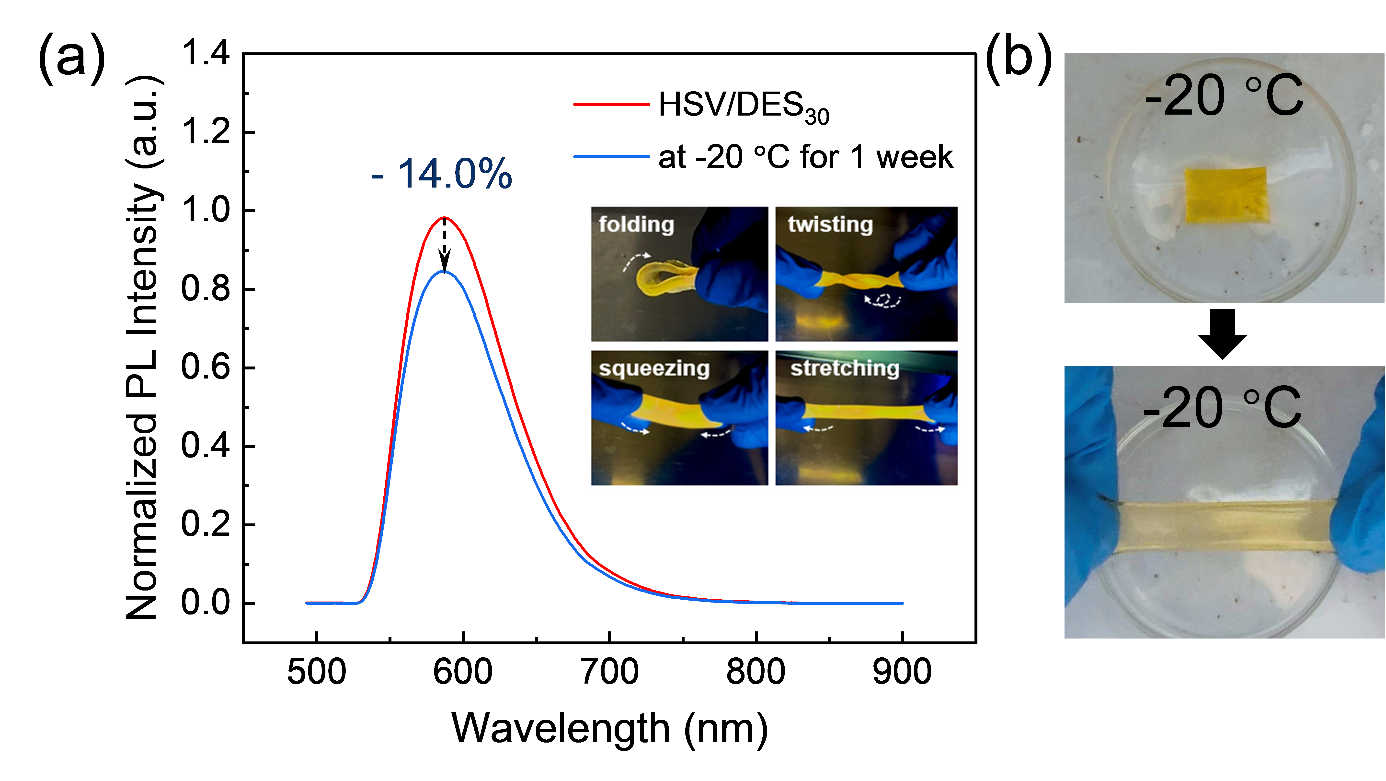


**Figure S16.** (a) PL spectra of HSV/DES_30_ eutectogel in -20 °C freezer for 1 week (λ_ex_ = 475 nm, B.W. = 2.5 nm). Insert photographs illustrating the flexibility, stretchability, and mouldability of the representative HSV/DES_30_ eutectogel after storage in -20 °C freezer for 1 week. (b) Photographs showing the stretchability of a representative HSV/DES_30_ eutectogel after ambient storage for three months followed by overnight cooling at -20 °C.

**Figure S17.** UV-Vis spectra of HSV/DES_30_ eutectogel recorded under -5 V for 30 min. Inserted photographs showing before and after electrochromic process.

**Figure S18.** UV-Vis spectra of St-MV-St solid recorded before and after 10 min UV irradiation.

**Figure S19.** Kinetic curve at 610 nm during photochromic process.

**Figure S20.** FTIR spectra of HSV/DES_30_ eutectogel before and after photochromism.

**Figure S21.** Kinetic decay curve at 610 nm during fading process.

**Figure S22.** The coloration/fading cycles of the HSV/DES_30_ eutectogel.

**Figure S23.** PL spectra of HSV/DES_30_ eutectogel before and after photochromism.


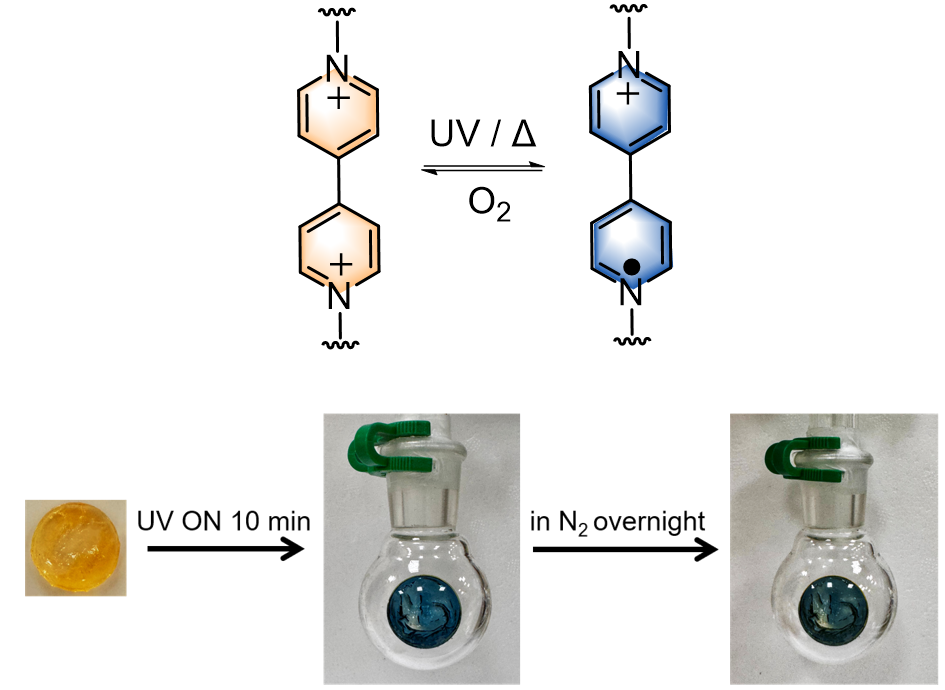


**Figure S24.** The photos of HSV/DES_30_ eutectogel (vacuum deaerated) after photochromism and put in N_2_ overnight.


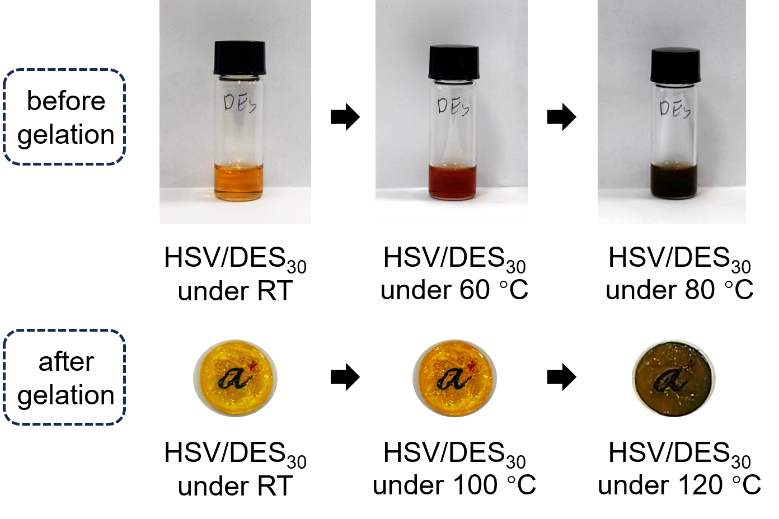


**Figure S25.** Photographs showing the thermochromic process under different temperature before and after gelation

**Figure S26.** UV-Vis spectra of HSV/DES_30_ eutectogel after heating to different temperature.

**Figure S27.** The spectral response curve at 610 nm during thermochromic process.


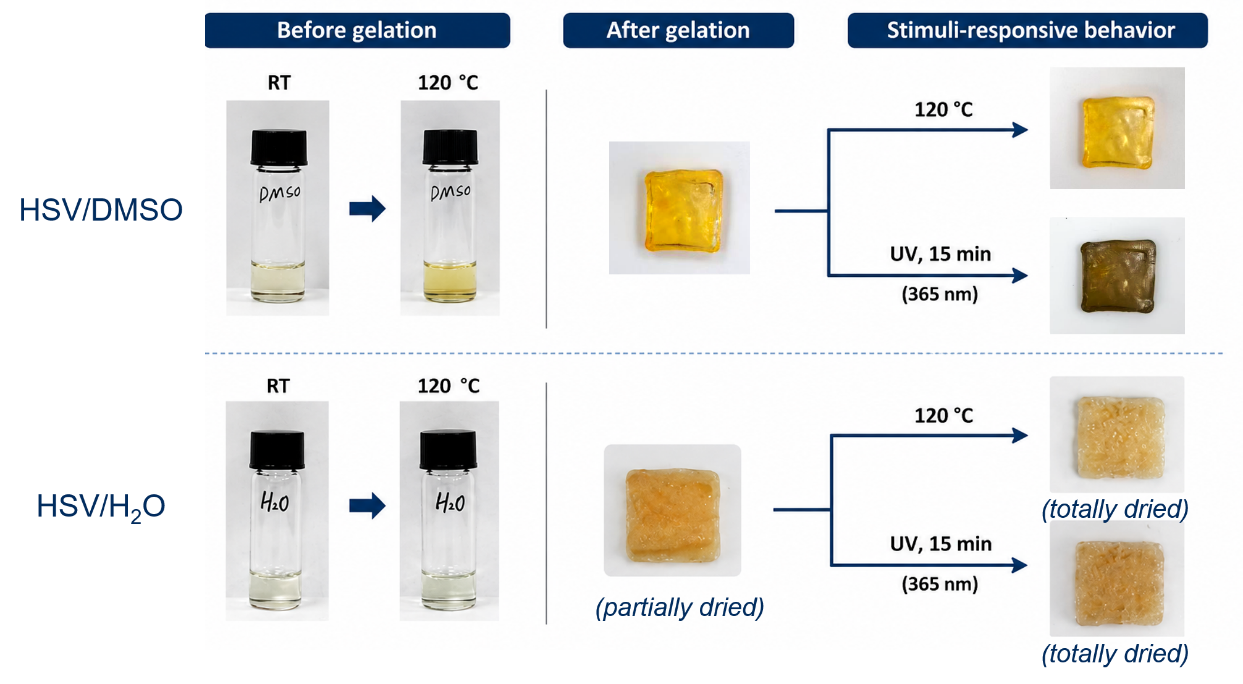


**Figure S28.** Control experiments showing the chromic behaviors of HSV/DMSO and HSV/H_2_O before and after gelation under thermal and UV stimulation.

**Figure S29.** UV-Vis spectra of HSV/DES_30_ eutectogel after heating to 120 ℃ and the fading process in air for 3 h.


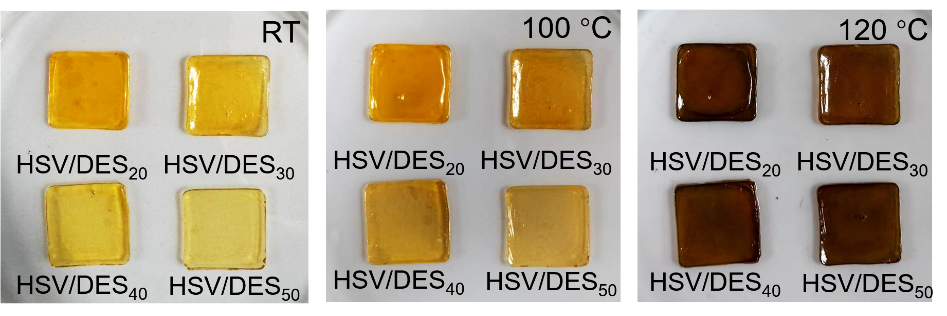


**Figure S30.** Photographs showing the thermochromic behavior of HSV/DES eutectogels with different NADES contents under RT, 100 °C, and 120 °C.

**Figure S31.** Response/recovery time curves of HSV/DES_30_ eutectogel under the strain of 10%.

**Figure S32.** Response/recovery time curves of HSV/DES_30_ eutectogel after photochromism under the strain of 10%.

**Figure S33.** Response/recovery time curves of HSV/DES_30_ eutectogel after thermochromism under the strain of 10%.

**Figure S34.** Relative resistance variation of the HSV/DES_30_ eutectogel under a strain continuously increased from 10 to 100% for 5 cycles.

**Figure S35.** Relative resistance variation of the HSV/DES_30_ eutectogel after photochromism under a strain continuously increased from 10 to 100% for 5 cycles.

**Figure S36.** Relative resistance variation of the HSV/DES_30_ eutectogel after thermochromism under a strain continuously increased from 10 to 100% for 5 cycles.

**Figure S37.** Relative resistance variation of the HSV/DES_30_ eutectogel under a strain from 10-100% for 3 times.

**Figure S38.** Relative resistance variation of the HSV/DES_30_ eutectogel

after photochromism under a strain from 10-100% for 3 times.

**Figure S39.** Relative resistance variation of the HSV/DES_30_ eutectogel after thermochromism under a strain from 10-100% for 3 times.

**Figure S40.** Relative resistance changes during consecutive hand stretching.


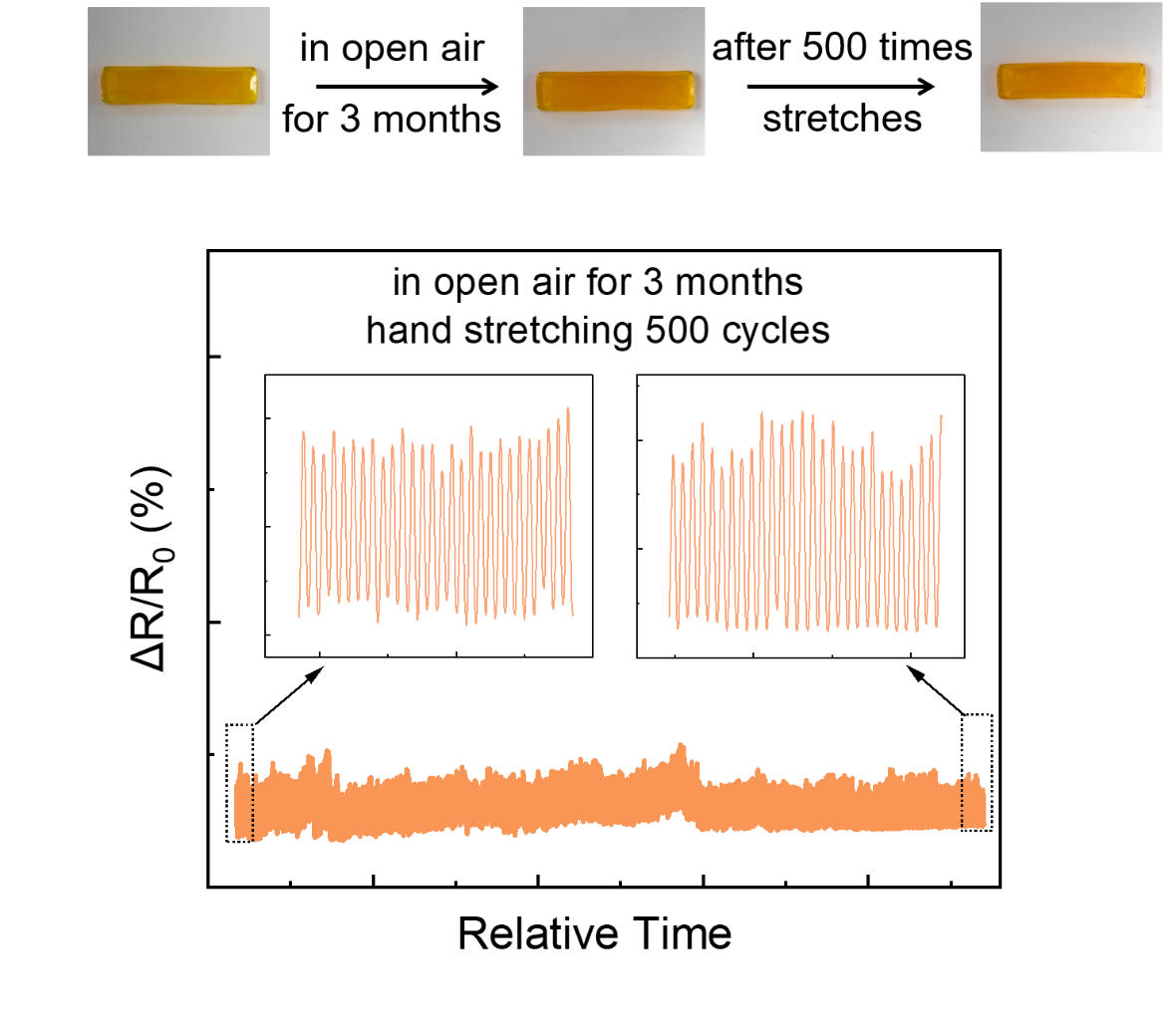


**Figure S41.** Relative resistance changes of the HSV/DES_30_ eutectogel during 500 consecutive hand-stretching cycles after storage in open air for 3 months.

**Table S2.** Comparison of key performance metrics of this work with recently reported viologen-based ionogels/hydrogels/eutectogels and related ionic gel sensors.

| **Ref** | **Viologen content** | **Stimulus response** | **Response time** | **Gauge factor** | **Strain sensing stability** | **Stimulus stability** |
| --- | --- | --- | --- | --- | --- | --- |
| [3] | 4 wt% [ImThV][TFSI] | Electrochromism (EC), electrofluorochromis  strain sensing | N.A. for strain-sensing, 1.8 – 2.0 s for EC | 1.2 – 2.3 | 500 cycles | 500 EC cycles |
| [4] | 0.1 wt% (SPr)_2_PhMeV | Electrochromism, strain sensing | N.A. for strain-sensing, EC within seconds | N.A. | N.A. | 700 EC cycles |
| [5] | EHP-Vinyl contents (16.6%, 33.3%, 66.6%) | Electrochromism, electrofluorochromism  strain sensing | 330 ms for strain-sensing,  39.1 s for EC | GF_1_ = 0.67,  GF_2_ = 0.98,  GF_3_ = 1.48 | 200 cycles | N.A. |
| [6] | SV/STV/ETV precursor ink at 5 mmol/L | Electrochromism | N.A. for stain-sensing, 25.7/28.1/22 s for EC | N.A. | N.A. | 5000 EC cycles |
| [7] | CT_1_-K_1_ with 11.4 wt% MVBBP for sensing | Strain sensing, temperature sensing | N.A. | GF_1_ = 4.02,  GF_2_ = 8.71 | 100 cycles | Stable repeated temperature sensing |
| [8] | HDPV/ASC molar ratio = 0.5/0.5 in iPU-ViO_0.5_-ASC_0.5_ | Electrochromism,  Pressure sensing, strain sensing, temperature sensing | EC within seconds, 15 s for temperature sensing, N.A. for strain sensing | GF_1_ = 0.34,  GF_2_ = 0.39 | 300 pressure cycles | 5000 EC cycles |
| [9] | 0.83 wt% relative to AM in PSE hydrogel | Electrochromism, pressure sensing | 4.2 s for EC, 16.8 s for pressure response | Pressure sensitivity = 358.1 kPa^−1^ | 10000 pressure cycles  400 bending cycles | 300 EC cycles |
| **This Work** | 0.2 mol% to HEA | Photochromism, photoluminescence, thermochromism, electrochromism, strain sensing | 1 s for photochromism, 0.31 s for strain sensing | GF_1_ = 0.0172,  GF_2_ = 0.0262 | 500 cycles | 20 cycles for photochromism |

# 4. Supplementary Videos

Supplementary Movie 1

Real-time demonstration of finger-bending detection using the HSV/DES_30_ eutectogel sensor. The sensor is mounted on a finger and subjected to repeated bending and releasing motions at different speeds. The device shows rapid, stable, and reproducible responses corresponding to the bending rate, highlighting its potential for real-time human motion monitoring.

Supplementary Movie 2

Real-time demonstration of wrist-bending detection using the HSV/DES_30_ eutectogel sensor. The sensor is attached to the wrist and undergoes repeated bending and relaxation during wrist motion. The device exhibits stable and reproducible responses corresponding to the bending deformation, demonstrating its capability for monitoring large-scale human joint movements.

Supplementary Movie 3

Real-time demonstration of physiological signal monitoring using the HSV/DES_30_ eutectogel sensor for heartbeat detection. The sensor is placed on the wrist to capture subtle pulse signals associated with the heartbeat. Clear and periodic signals corresponding to the pulse are observed, highlighting the high sensitivity of the sensor for detecting weak physiological signals.

# 5. Reference

[1] F. Mohd Fuad, M. Mohd Nadzir, *J Mol Liq* **2022**, *360*, 119392.

[2] C. S. Y. Tan, J. Liu, A. S. Groombridge, S. J. Barrow, C. A. Dreiss, O. A. Scherman, *Adv Funct Mater* **2018**, *28*, 1702994.

[3] Y. Zhang, M. Guo, G. Li, X. Chen, Z. Liu, J. Shao, Y. Huang, G. He, *CCS Chemistry* **2022**, *5*, 1917-1930.

[4] Z. Han, H. Yuan, H. Zhang, Y. Zhang, J. Lv, X. Zhang, Z. Wang, N. Li, C. Liang, N. Yan, M. Maximov, Y. Huang, G. He, *CCS Chemistry* **2024**, *7*, 854-866.

[5] J. Zhao, Y. Zhou, Y. Wang, Q. Liang, X. Ma, X. Jia, D. Chao, *Acs Appl Mater Inter* **2023**, *15*, 38821-38832.

[6] X. Luo, R. Wan, Z. Zhang, M. Song, L. Yan, J. Xu, H. Yang, B. Lu, *Adv Sci* **2024**, *11*, 2404679.

[7] R. Zhang, C. Liu, C. Wei, Y. Wang, F. Li, Z. Zhang, J. Qu, N. Qing, L. Tang, *J Mater Chem A* **2023**, *11*, 8320-8329.

[8] Q. Fan, X. Jiang, Y. Zhang, L. Ge, W. Yang, J. Li, Y. Li, K. Wu, X. Wu, Q. Zhang, Y. Li, C. Hou, K. Li, J. Yu, Y. Wang, H. Wang, *Small* **2025**, *21*, 2412798.

[9] T. Sun, W. Yue, T. Sun, H. Niu, Y. Li, *Adv Funct Mater* **2025**, *35*, 2503841.
